# Interaction of the NRF2 and p63 transcription factors promotes keratinocyte proliferation in the epidermis

**DOI:** 10.1093/nar/gkab167

**Published:** 2021-03-25

**Authors:** Svitlana Kurinna, Kristin Seltmann, Andreas L Bachmann, Andreas Schwendimann, Lalitha Thiagarajan, Paulina Hennig, Hans-Dietmar Beer, Maria Rosaria Mollo, Caterina Missero, Sabine Werner

**Affiliations:** Division of Cell Matrix Biology and Regenerative Medicine, FBMH, University of Manchester, M13 9PT, United Kingdom; Department of Biology, Institute of Molecular Health Sciences, ETH Zurich, 8093 Zurich, Switzerland; Department of Biology, Institute of Molecular Health Sciences, ETH Zurich, 8093 Zurich, Switzerland; Department of Biology, Institute of Molecular Health Sciences, ETH Zurich, 8093 Zurich, Switzerland; Division of Cell Matrix Biology and Regenerative Medicine, FBMH, University of Manchester, M13 9PT, United Kingdom; Department of Dermatology, University Hospital Zurich, 8006 Zurich, Switzerland; Department of Dermatology, University Hospital Zurich, 8006 Zurich, Switzerland; CEINGE Biotecnologie Avanzate, Naples, Italy, University of Naples Federico II, 80131 Naples, Italy; CEINGE Biotecnologie Avanzate, Naples, Italy, University of Naples Federico II, 80131 Naples, Italy; Department of Biology, Institute of Molecular Health Sciences, ETH Zurich, 8093 Zurich, Switzerland

## Abstract

Epigenetic regulation of cell and tissue function requires the coordinated action of transcription factors. However, their combinatorial activities during regeneration remain largely unexplored. Here, we discover an unexpected interaction between the cytoprotective transcription factor NRF2 and p63- a key player in epithelial morphogenesis. Chromatin immunoprecipitation combined with sequencing and reporter assays identifies enhancers and promoters that are simultaneously activated by NRF2 and p63 in human keratinocytes. Modeling of p63 and NRF2 binding to nucleosomal DNA suggests their chromatin-assisted interaction. Pharmacological and genetic activation of NRF2 increases NRF2–p63 binding to enhancers and promotes keratinocyte proliferation, which involves the common NRF2–p63 target cyclin-dependent kinase 12. These results unravel a collaborative function of NRF2 and p63 in the control of epidermal renewal and suggest their combined activation as a strategy to promote repair of human skin and other stratified epithelia.

## INTRODUCTION

The development and regeneration of epithelial tissues is a highly organized process, which is at least in part regulated at the level of transcription (1). DNA binding to regulatory elements (RE) and transcriptional activity of p63 family members are indispensable for the development and continuous renewal of the epidermis and other stratified epithelia (2). The deltaNp63alpha isoform, called here p63 for simplification, is abundant in the epidermis and supports an undifferentiated, proliferative state during keratinocyte lineage specification (3). The nuclear factor erythroid 2-related factor 2 (NFE2L2; NRF2) is a ubiquitously expressed cytoprotective transcription factor (TF) with demonstrated functions in wound repair and skin cancer (4). Under unchallenged conditions, the NRF2 inhibitor protein KEAP1 retains NRF2 in the cytoplasm and also mediates its continuous proteasomal degradation. However, a small pool of NRF2 escapes this mechanism, resulting in nuclear translocation, dimerization with small Maf proteins and binding to antioxidant response elements (AREs) in promoters or enhancers of target genes. Further activation of NRF2 occurs through conformational changes of KEAP1, which are mainly induced by the covalent coupling of electrophilic molecules to this protein. These changes result in the weakening of the NRF2–KEAP1 interaction and stabilization of NRF2. As a consequence, the NRF2 turnover is modified and newly synthesized NRF2 is stabilized and accumulates in the nucleus, resulting in upregulation of NRF2 target gene expression (5).

While *Nrf2* knockout mice do not have obvious epidermal abnormalities (6), loss of this gene in combination with other genes relevant for the epidermal barrier is deleterious (7). The overactivation of Nrf2 in a normal epidermis is, however, also not desirable, since the expression of a constitutively active Nrf2 (caNrf2) mutant in keratinocytes of transgenic mice caused epidermal hyperplasia, hyperkeratosis and hair follicle cyst formation (8,9). Similar to the function of p63 in the differentiation of stratified epithelia, activation of Nrf2 in the esophagus regulates progenitor cells through yet unknown mechanisms (10,11). The loss of Nrf2 inhibited the mobilization of p63-positive stem cells in the lung, a key event for the repair of this tissue and prevention of fibrosis (12). These studies suggest that both Nrf2 and p63 are important for epithelial cell proliferation and/or differentiation, but a potential functional interaction of p63 and NRF2 has as yet not been demonstrated. Here, we use available and new chromatin immunoprecipitation and sequencing data to test the combinatorial binding of human NRF2 and p63 at a genome-wide level to elements designated RE-AREs. We discover that p63 and NRF2 interact on the RE-AREs in enhancers and promoters of selected genes in primary human keratinocytes and we unravel the underlying molecular mechanism. The combined activity of NRF2 and p63 is functionally relevant, since it promotes keratinocyte proliferation and thus the regenerative potential of the skin.

## MATERIALS AND METHODS

### Mouse maintenance and experimentation

Mice were maintained under specific pathogen-free conditions and obtained food and water *ad libitum*. Mouse maintenance and experiments with mice were approved by the local veterinary authorities (Kantonales Veterinäramt Zürich, Switzerland). Mice expressing caNrf2 or dnNrf2 in keratinocytes were previously described and used for ChIP analyses of epidermal lysates (7,13).

### Human skin samples

The human foreskin was obtained anonymously from consenting parents in the context of the University of Zurich biobank project, approved by the local and cantonal Research Ethics Committees.

### 2D cultures of human keratinocytes and 3D skin equivalents

Human primary keratinocytes were isolated from the foreskin of two healthy boys using dispase and expanded on feeder layer fibroblasts. Early passage (2-3) cells were used in all experiments. To generate 3D skin equivalents, keratinocytes were seeded onto collagen scaffolds and allowed to grow and differentiate for 7 days at the air–liquid interface (14). The scaffolds with epidermal layer were collected, fixed and sectioned. Undifferentiated keratinocytes of the basal epidermal layer are cytokeratin 14 positive (K14), do not express K10, and proliferate in sub-confluent submerged 2D cultures in the presence of growth factors (GF) (15,16).

### CRISPR-Cas9-mediated knockout of KEAP1 in keratinocytes and compound treatment

Primary human keratinocytes were transduced with the lentiCRISPRv2 plasmid (Addgene), which includes an expression cassette for Cas9 and encodes two different sgRNAs targeting KEAP1 as previously described (17,18).

The CDK12 inhibitor THZ531 (MedChemExpress), and the NRF2 activating compounds sulforaphane (SFN; Sigma) and tert-butylhydroquinone (tBHQ; Sigma) were added to primary human keratinocytes as described in the figure legends.

### miR *in situ* hybridization

Based on previous work on miR *in situ* hybridization analysis with fluorescently labeled LNA probes (19), we developed a modified miR-29 *in situ* hybridization protocol. Briefly, cryopreserved human foreskin biopsies were sectioned (14 μm) and fixed with 4% paraformaldehyde (PFA). miR-29a and scrambled sequences (19), synthesized and Cy2-labeled by RiboTask, were hybridized at 63°C (miR-29a) or 55°C (scrambled). Sections of the mouse hippocampus, which expresses high levels of miR-29a, were used as a positive control, whereas skin sections from *miR29ab1* knockout mice hybridized with the miR-29a probe, as well as human skin sections hybridized with the scrambled probe, served as negative controls to find the optimal temperature for hybridization and washing steps (data not shown). *In situ* hybridization was followed by immunofluorescence staining with antibodies against pNRF2 and p63 (see below).

### siRNA transfection, RNA isolation, qRT-PCR and miR expression analysis

siRNAs targeting deltaNp63α (referred here as p63) (5′-CACCCUUAUAGUCUAAGACUA) or NRF2 (5′-GAGAAAGAAUUGCCUGUAA) were used for transfection of primary human keratinocytes using INTERFERin (Polyplus). Total RNA was isolated from keratinocytes using Trizol reagent (Invitrogen/Thermo Fisher Scientific) and used for cDNA synthesis with random hexamer primers for measuring the primary transcript levels. TaqMan miR-specific assays (Ambion/Thermo Fisher Scientific) were used to measure mature miR levels. qRT-PCR was performed with SensiFast cDNA Synthesis Kit (Bioline) using the manufacturer’s protocol, followed by PCR using *RPL27* as reference gene and primers listed in Supplementary Table S1.

### Chromatin immunoprecipitation (ChIP), re-ChIP and ChIP-seq

ChIP experiments were performed using epidermis from adult (8 weeks old) mice expressing caNrf2 in keratinocytes (K5-CMVcaNrf2 mice) and control mice as described previously (13). The hairs on the back were shaved, fat from the back and tail skin was removed, and the epidermis was peeled off following dispase treatment and snap-frozen in liquid nitrogen. For ChIP and re-ChIP from human keratinocyte lysates, cells were first grown to confluency and where indicated, treated with 5 μM SFN for 3 h. They were scraped off in cold PBS containing protease, phosphatase, and deacetylase inhibitors and snap-frozen. For re-ChIP, chromatin was eluted in NaHCO_3_-SDS buffer following the first IP, and the supernatant was collected from at least two tubes containing the same antibody. Samples were kept on ice and diluted 1:10 with pooling buffer (25 mM Tris pH 8, Triton-X100 (1% (v/v)), 5 mM EDTA, 150 mM NaCl, phosphatase inhibitors, HDAC inhibitors, dH_2_O). The following antibodies were used for the second IP: rabbit or sheep IgG (5 μl), total NRF2 (Santa Cruz; C-20 ChIP-grade, 2.5 μl), p63 (Santa Cruz; H-129, 25 μl, and 4A4 clone, Abcam), Histone 3 (H3) (Abcam, ChIP-grade, 4 μl) and H3K4me2 (Active Motif; ChIP-grade, 10 μl). H3K4me2 as the first antibody and H3 as the second antibody used in a parallel re-ChIP experiment served as a positive control. ChIP-seq was performed in triplicates using lysates from primary human keratinocytes treated with 5 μM SFN or vehicle for 3 h in the absence of growth factors and using an antibody against total NRF2 (Active Motif; ChIP-grade, 10 μl). 23.4/5.2 and 23.8 /7.9 million reads were mapped to GRChg38 (Bowtie 2, default parameters) from pulled NRF2/input and NRF2SFN/input samples, respectively. Peak calling was done using MASC2 with default parameters and band width of 300 bp, where only the peaks with *P* value < 0.05 were requested.

### Luciferase reporter assays

RE-ARE sequences were cloned into pGL3 and pGL4 firefly luciferase reporter vectors (Promega) and used for co-transfection with a control reporter vector containing the Renilla luciferase gene to normalize for transfection efficiency (Promega). Plasmids coding for the Flag-tag only, Flag-tagged wt deltaNp63alpha, R304Q, and L514F mutants of deltaNp63alpha (20), CMV-caNrf2, HA-tagged wt and dominant-negative Nrf2 (dnNrf2) were described previously (7,13). The Promega Dual Glo system was used to measure Firefly and Renilla luciferase activities 24–48 h after transfection.

### Immunofluorescence staining

Sections from cryo-preserved human foreskin or 3D human skin equivalents and freshly isolated primary human keratinocytes were used. Antibodies detecting nuclear (active) phospho-NRF2 (S40) (Abcam, EP1809Y) or p63 (Abcam, clone 4A4) were applied overnight following 4% PFA fixation of the specimens. K10 and K14 antibodies were from DAKO, and the Ki-67 antibody (SolA15) was from eBioscience (Thermo Fisher Scientific). Fluorescently labeled secondary antibodies (Jackson ImmunoResearch) were used. Nuclei were counterstained with DAPI. Cells were counted using ImageJ (National Institutes of Health). p63- and pNRF2-stained sections were analyzed by confocal microscopy. Deconvolution of confocal images was performed using Leica software.

### Proximity ligation assay (PLA)

Proximity ligation assay (PLA) was performed by applying the pNRF2 and p63 antibodies as described for fluorescence microscopy, followed by ligation of probes specific for each antibody using a PLA Kit (Sigma). The probe-specific signal was detected by confocal microscopy and quantified following deconvolution in Imaris (Oxford Instruments).

### SDS-PAGE and Western blot analysis

Total cell lysates were separated by SDS-PAGE and analyzed by western blotting using antibodies against p63 (Santa Cruz; H-129), NRF2 (Santa Cruz; C-20), K10 (DAKO) and β-actin (Sigma).

### Genome-wide identification of RE-ARE enhancers

We generated hypothetical sequences of chromosomes with randomly distributed RE-ARE pairs with the same number of RE and ARE motifs as in the real genome (Supplementary File S1). Next, we used an R script (Supplementary File S2) to pair REs with the nearest AREs to compute the distances between RE and ARE motifs in the experimental (real) genome versus distances between randomly distributed RE and AREs (Supplementary Table S2). Using the same random versus experimental (real) RE-AREs, we calculated distances of RE-ARE pairs to the nearest TSS genome-wide with an R script (Supplementary File S3). We determined the RE-ARE-TSS distances in the genome using either hg19 TSS or miRBase (mirbase.org) TSS positions and calculated the distance from each TSS to the closest RE-ARE (Supplementary Table S3). In this way, we provided a standardized format for the information associated with each RE-ARE pair, and computed the coordinates of RE-ARE elements to functionally annotate each pair.

### Gene annotations and gene ontology (GO) analysis

To identify RE-ARE pairs below H3K27ac and p63 peaks in proliferation-competent and differentiated keratinocytes, we used a Python script (Supplementary File S4). The peak of H3K27ac was defined when an enrichment in mapped reads from H3K27ac ChIPseq was found within 300 bp from the center of the peak. A pair was considered to be below a peak if the start and the end of each motif were fully within the peak range. The extended output of this script includes peaks containing only one motif. This was used to assess all RE-ARE pairs, which are below a p63 peak (Supplementary Table S4). This subset was then combined in R with RE-ARE-containing H3K27ac peaks to calculate percentages of RE-AREs associated with p63-H3K27ac peaks. Analysis for peaks containing at least one RE-ARE pair was performed using GREAT (http://bejerano.stanford.edu/great/public/html/). Each H3K27ac peak containing a RE-ARE was associated with the closest gene within 100 kb. When two RE-ARE pairs were below one peak, the gene was assigned in duplicate. The resulting GO labels were sorted by the lowest binomial FDR values into two GO-groups: biological processes and cellular components. Some categories were binned into summary-categories with close FDR values and nearly identical function. In differentiating keratinocytes (DK), the following categories were combined: cell death: cell death, death. We used R to compare two sets of H3K27ac peaks containing RE-ARE pairs in KP and DK. We obtained subsets of RE-ARE-containing H3K27ac peaks unique and common for KP or DK. The detailed gene list associated with a GO-label can be found in Supplementary Tables S5–S9.

### Analysis of functional categories using GREAT

The search for unique and common RE-ARE-containing H3K27ac peaks for KP and DK was then performed as described in the previous section. Summary categories in unique DK are the following: small molecule metabolic process: small molecule metabolic process, organophosphate metabolic process; cellular localization: cellular localization, macromolecular localization; catabolic process: catabolic process, organic substance catabolic process; cell death: cell death, death. For common peaks, the following categories were combined: regulation of programmed cell death: regulation of programmed cell death, regulation of apoptotic processes. The detailed gene list associated with a GO-label can be found in the Supplementary Information (unique KP: Supplementary Table S7, unique DK: Supplementary Table S8, common: Supplementary Table S9). Sequences of RE-ARE pairs for MEME analysis (http://meme-suite.org/tools/meme) were obtained using a Python script (Supplementary File S5).

### TF-DNA model generation

DNA-binding domains of p63 and the nucleosome core particle, small Maf proteins (dimerization partners of Nrf2 (21), and Skn-1 (*Caenorhabditis elegans* ortholog of Nrf2) were used to model the Nrf2–p63 interactions on RE-ARE sequences in PyMOL v1.3 software with Tcl-Tk GUI features. The structures used are taken from NCBI PDB with the following IDs: p63 DBD tetramer 3QYN, p63 DBD monomer: 2RMN; Skn-1 DBD: 1SKN-1; MafB DBD: 2WT7; spacer DNA 2L8Q; nucleosome: 5AV5. Images and movies were generated directly in PyMOL.

### Statistical analysis

Statistical analysis of the distributions of random and experimental RE-ARE pairs was done using a Kolmogorov–Smirnov test in R v3.2.5. Statistical analysis for ChIP was performed using paired Student’s *t*-test when samples followed Gaussian distribution; otherwise, Mann–Whitney *U* test was applied. The cell count and RNA expression were analyzed by ANOVA and unpaired Student’s *t*-test where applicable (GraphPad Software Inc). Luciferase assays were analyzed in R.

## RESULTS

### p63-NRF2 regulatory elements are located in active enhancers of proliferation-competent cells

To determine a potential cross-talk of NRF2 and p63, we used human keratinocytes as a model system and determined in a computational approach if both TFs may influence each other on the genome. TFs bind specific DNA consensus sequences located in regulatory elements (REs) of promoters and enhancers of a gene or a group of genes (22,23). The NRF2 and p63 DNA-binding sequences (ARE and RE, respectively) were IUPAC-coded based on the sequences identified by ChIP-seq analysis with NRF2 (24) or p63 (25) (Supplementary Figure S1A). The locations of p63 REs and NRF2 AREs were extracted from a genome-wide analysis of human genome assembly GRCh37 using a Python script (Supplementary File S1 and Supplementary Figure S1A). As a control for non-random RE-ARE distance distribution, a total number of REs (90 798) and AREs (725 362) discovered genome-wide were distributed proportionally to the length of the chromosome as compared to the length of the genome. When we estimated the distance between all REs and AREs genome-wide, we found a non-random spacer length enrichment within a 2000 bp window with the *P* value 2.2 × 10^−16^ (Supplementary Figure S1B) suggesting that REs and AREs may be located close to each other, allowing p63 and NRF2 to interact. The minimal distance that allows p63-NRF2 binding to the RE-ARE without a steric hindrance is >7 bp regardless of the relative positions of p63 REs and NRF2 AREs along the DNA (Supplementary Figure S1C). These results predict that p63 and NRF2 interact genome-wide and in a DNA-facilitated manner.

**Figure 1.**
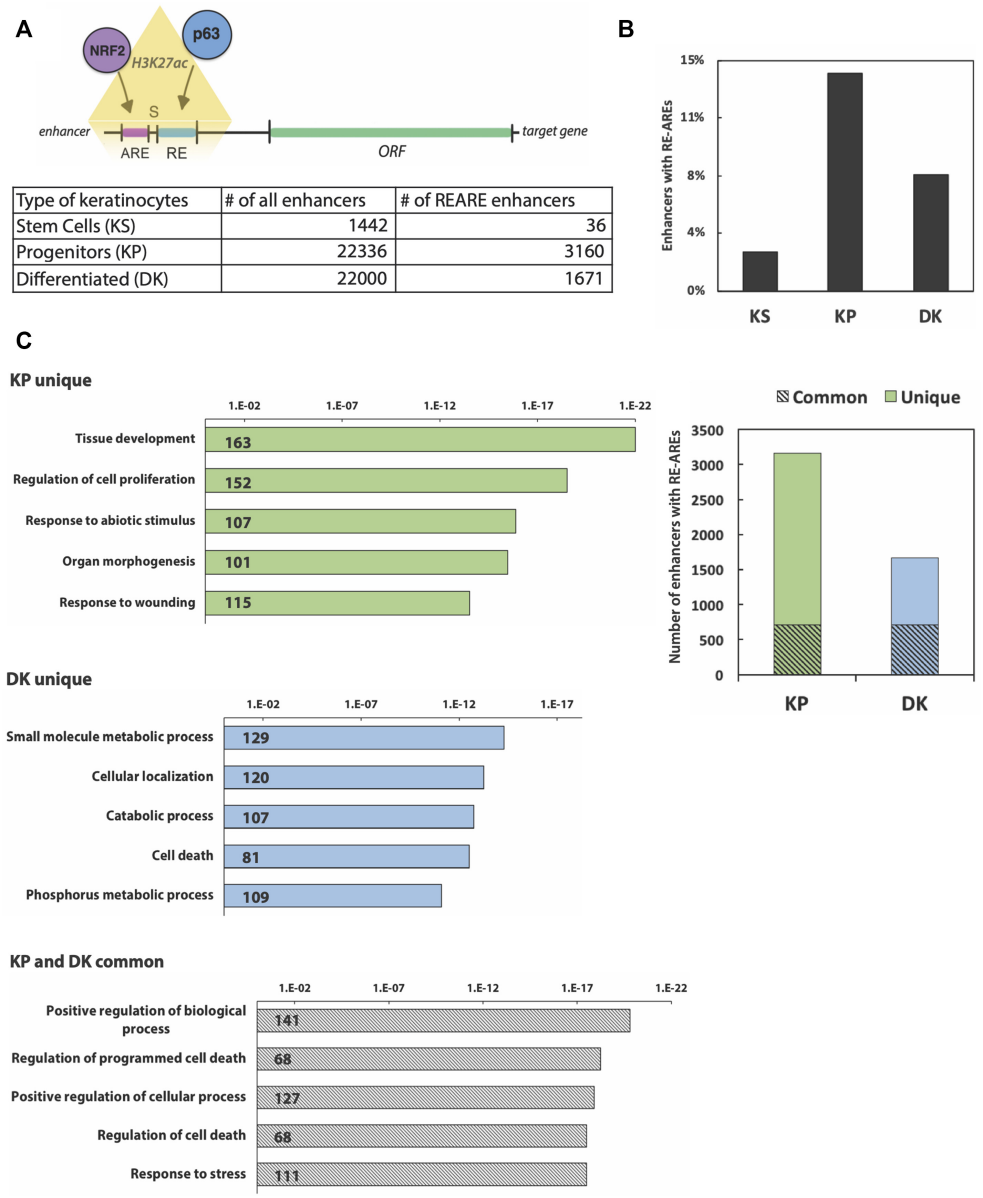
RE-AREs are present in active enhancers in human keratinocytes with proliferative potential. (**A**) NRF2-binding ARE and p63-binding RE sequences (RE-AREs) were identified and quantified below H3K27ac peaks marking active enhancers in keratinocyte stem cells (KS), and in proliferation-competent/undifferentiated (KP) and differentiated keratinocytes (DK). For the nomenclature see reference (16). (**B**) The number of RE-ARE enhancers from the table in (A) is presented as percentage of all H3K27ac-positive enhancers. (**C**) H3K27ac peaks containing RE-ARE pairs in KP and DK. Obtained subsets of RE-ARE-containing H3K27ac peaks were unique or common for KP or DK. GREAT analysis for unique and common RE-ARE-containing H3K27ac peaks for KP and DK was then performed by associating each H3K27ac peak containing RE-ARE with the closest gene within 100 kb. The resulting GO labels were sorted by the lowest binomial FDR values into GO-groups.

To correlate RE-ARE positions with known regulatory DNA regions, we used published p63 and histone H3K27ac ChIP-seq data obtained from primary human keratinocytes at different stages of differentiation (15). Acetylated H3K27 residues mark promoters and enhancers in keratinocytes (15,16) and thus can narrow down predicted RE-AREs to active regulatory elements (Figure 1A). Comparison of coordinates of the RE-ARE pairs to positions of H3K27ac peaks in proliferation-competent (designated in the original study as keratinocyte stem (KS) and precursors (KP) (26)) revealed that KPs have more active, H3K27ac RE-AREs (Figure 1B). Gene ontology (GO) analysis of the genes with RE-ARE-containing enhancers revealed categories of genes relevant for tissue development and cell proliferation in KPs, shifting to metabolic processes in differentiated cells (DK, Figure 1C). Common pathways in KP and DK possibly controlled by RE-ARE enhancers in keratinocytes include regulation of cell death and response to stress (Figure 1C) and are consistent with functional annotation of all common enhancers found in KPs and DKs. Taken together, these data predict a functional relevance of RE-AREs in proliferation-competent keratinocytes.

### p63 and NRF2 interact in the nucleus of primary human keratinocytes

**Figure 2.**
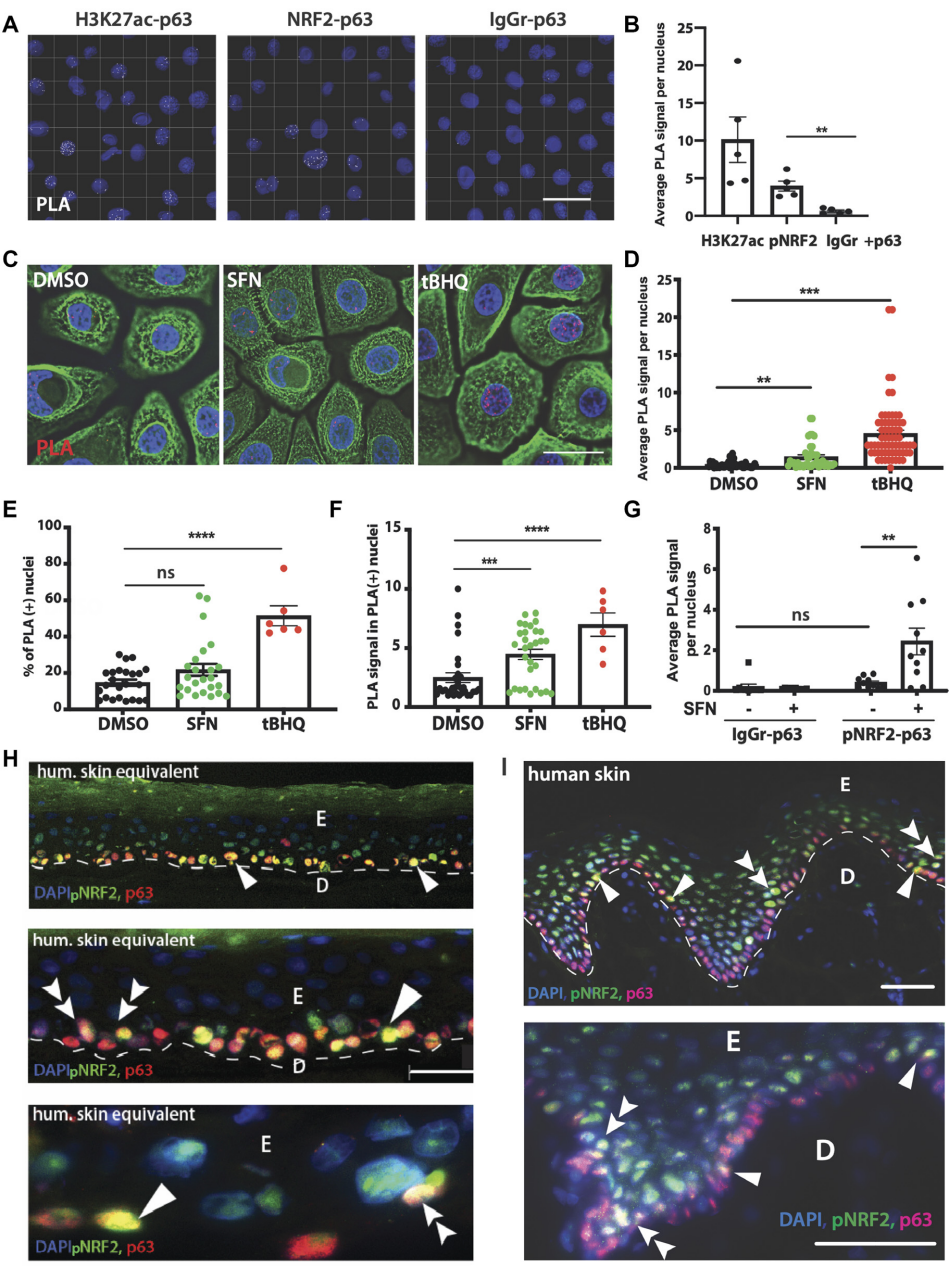
p63 and activated NRF2 are in close proximity to each other in the nucleus. (**A–G**) PLA using p63, pNRF2 and control antibodies. When the antibodies detect epitopes located in close proximity, the attached probes interact and produce a fluorescent signal (far-red, shown as white (A) or red (C) dot). (A) Representative images showing PLA signals in primary human keratinocytes upon staining with antibodies against pNRF2 and p63 (middle panel). Antibodies against H3K27ac and p63 were used as a positive control for PLA (left panel). Unspecific rabbit IgG was used as a negative control in combination with mouse p63 IgG (IgGr-p63) (right panel); *N* = 3, approximately 50 nuclei were counted in five fields of view for each biological replicate. The number of nuclei with PLA signal was divided by the total number of nuclei; scale bars: 50 μm. (B) Average PLA signal per nucleus calculated as total PLA puncta divided by the number of all nuclei in the field. At least 10 fields with 50–100 nuclei were quantified per treatment in (B), (D), (E) and (F). (C) Representative images from PLA assays (red) in primary human keratinocytes treated with SFN (5 μM, 3 h) or tBHQ (25 μM, 3 h), counterstained with antibodies against keratin 14 (green) and with DAPI (blue), showing p63–pNRF2 interactions; scale bar: 20 μm. (D) PLA signal calculated as in (B). (E) Percentage of PLA-positive nuclei. (F) Average number of PLA signals in PLA-positive nuclei only. (G) Primary human keratinocytes treated with DMSO or SFN, PLA signal calculated as in (B). (**H**) 3D cultures of primary human keratinocytes were air-lifted for 7 days, allowing formation of differentiated layers. Cultures were fixed, sectioned and stained with antibodies detecting pNRF2 (active nuclear NRF2 (green)), and p63 (red). Nuclei were counterstained with DAPI (blue); scale bars: 100 μm (upper), 50 μm (middle) and 10 μm (lower panel). (**I**) Sections of human foreskin were stained with antibodies against pNRF2 (green) and p63 (red). Nuclei were counterstained with DAPI (blue). Double-positive cells among undifferentiated keratinocytes of the basal layer are indicated with single arrowheads. Early suprabasal keratinocytes expressing both NRF2 and p63 are indicated with double-arrowheads; scale bar: 100 μm. Bars indicate mean ± SEM. ns: non-significant; **P < 0.01, ***P < 0.001, ****P < 0.0001 (Mann–Whitney *U* test).

To test if p63 and NRF2 physically interact *in vivo*, we performed proximity ligation assays (PLA) in primary human keratinocytes using antibodies against total p63 and S40-phosphorylated NRF2, which is considered as the active, nuclear form (27). PLA revealed the interaction between p63 and H3K27ac (positive control) and also with pNRF2 (Figure 2A and B). Treatment with the NRF2 activating compounds sulforaphane (SFN) (21) or tBHQ significantly increased the number of PLA puncta (Figure 2C and D), most likely due to stabilization of NRF2 and its accumulation in the nucleus. tBHQ also increased the number of nuclei with a PLA signal (Figure 2E). Both compounds increased the intensity of pNRF2-p63 signals per nucleus (Figure 2F). Importantly, this happened within a short treatment period (3 h) and while cells continued to proliferate (Supplementary Figure S2A), suggesting that pNRF2–p63 interactions are initiated immediately upon stabilization of NRF2 and that this process is compatible with the proliferative potential of primary cells. Even though we could only reliably detect the NRF2–p63 interactions in SFN-treated, but not in untreated cells (Figure 2G), we detected nuclei positive for both pNRF2 and p63 in a 2D differentiation model of primary keratinocytes (Supplementary Figure S2B) and in a 3D organotypic skin model (Figure 2H) (14,28). pNRF2 was detected in subconfluent, proliferating cells and during differentiation, whereas p63 expression increased at the point of growth factor withdrawal (Supplementary Figure S2B) (15). Co-localization of p63 and pNRF2 was detected in the nuclei of basal and lower suprabasal keratinocytes in the 3D human skin equivalents (Figure 2H) and in basal and lower suprabasal layers in rete ridges of the human epidermis (Figure 2I and Supplementary Figure S2C). Together, these results demonstrate that p63 and NRF2 interact in the nuclei of human keratinocytes during proliferation and early differentiation and that NRF2-activating compounds promote this interaction.

### Binding of p63 to the NRF2-occupied RE-ARE augments transcriptional activation

To determine if NRF2 and p63 jointly regulate target genes, we first checked if known direct targets of NRF2 in keratinocytes (9,29) are also targets of p63 and contain a predicted RE-ARE. Indeed, the gene encoding the precursor of microRNA (miR)-29a and miR-29b1 is a direct and functionally relevant NRF2 target in mouse and human keratinocytes (13) and also a target of p53 (30), which can bind to the same response elements as p63 (31). Immunostaining combined with *in situ* hybridization showed that nuclear pNRF2 correlates with the miR-29a signal in suprabasal layers, and p63/miR29a-positive cells were also present in this layer (Supplementary Figure S3A). This is functionally relevant, since siRNA-mediated knockdown of p63 significantly suppressed the levels of mature miR-29a and of the primary transcript of the *MIR29AB1* gene-pri-(miR29a/b1) (Supplementary Figure S3B and S3C). This was even more pronounced upon simultaneous knockdown of p63 and NRF2, indicating that the transcriptional activation of the *MIR29AB1* gene relies on their joint activity (Supplementary Figure S3C).

**Figure 3.**
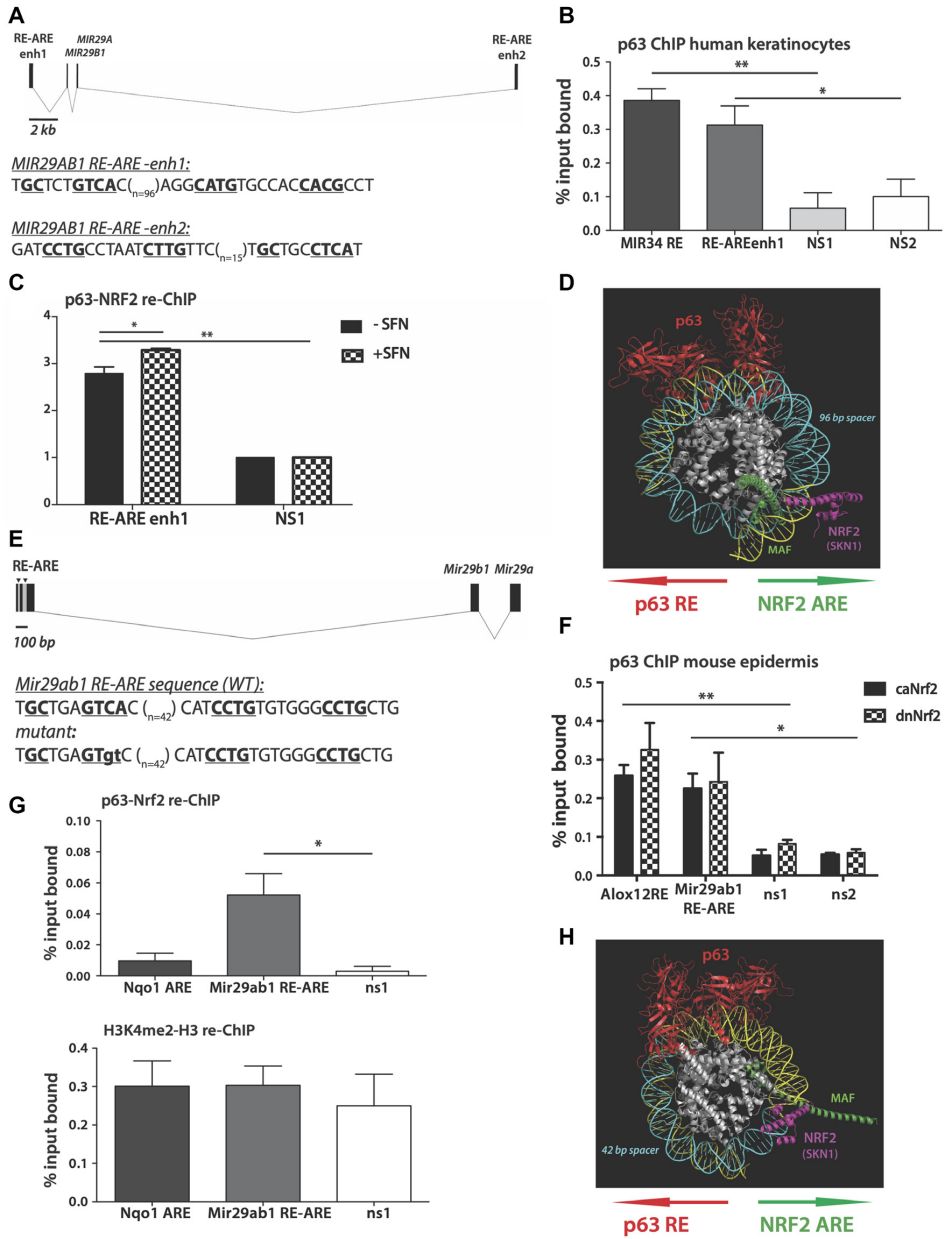
Simultaneous DNA binding of p63 and NRF2 activates *Mir29ab1* transcription. (**A**) Positions of the p63 RE and NRF2 ARE (RE-ARE) relative to miR-29a and miR-29b1 coding regions in the human genome. Core nucleotides of RE-ARE sequences are underlined and highlighted in bold. Number of base pairs forming the spacer *n* is indicated. (**B**) p63 ChIP using lysates of HaCaT keratinocytes showing significant binding of p63 to the control gene (*MIR34*) (20) and to one of the *MIR29AB1* RE-AREs. Non-specific RE-void regions (ns1 and ns2) serve as a negative control. (**C**) Sequential re-ChIP with p63 and NRF2 antibodies using lysates from confluent, but undifferentiated primary human keratinocytes treated or not with the NRF2 activating compound sulforaphane (SFN); *N* = 3. (**D**) Structural model of NRF2 and p63 binding to DNA. The human *MIR29AB1* RE-ARE-up sequence is shown as a DNA backbone in yellow on a core nucleosome. Histones (white) are shown with the DNA-binding domains (DBDs) of a p63 tetramer (red) and an NRF2/Skn-1-small MAF dimer (magenta and green correspondingly). Note that the spacer (blue) separates RE and ARE sequences under p63 and NRF2/Skn-1-MAF DBDs and thus allows for simultaneous binding of p63 and NRF2 on the nucleosome. (**E**) Positions of the RE-ARE relative to the miR-29a and miR-29b1 coding regions in the mouse genome. Core nucleotides of p63 RE and ARE sequences are underlined and highlighted in bold. Number of base pairs forming the spacer *n* is indicated. Mutated base pairs (in small-case letters) for later experiments are shown below the wt sequence. (**F**) p63 ChIP using epidermal lysates of transgenic mice expressing constitutively active (caNrf2) or dominant-negative (dnNrf2) Nrf2 mutant in keratinocytes. *Alox12* p63 RE served as a positive control (33); primers hybridizing at least 2 kb away from *MiR29ab1* and *Alox12* REs and void of p63 REs (ns1 and ns2) served as a negative control; *N* = 5. (**G**) Top panel: re-ChIP with p63 and Nrf2 antibodies using epidermal lysates of caNrf2-transgenic mice shows simultaneous binding of p63 and Nrf2 to RE-ARE of *Mir29ab1*. Nrf2-only bound to an ARE of the classical Nrf2 target *Nqo1* and a non-specific (ns) region 2 kb away from *Mir29ab1* RE-ARE served as negative controls; *N* = 5. Lower panel: re-ChIP with an antibody against H3K4me2 followed by an antibody against total histone H3 shows presence of the H3K4me2 mark in *Nqo1* ARE regions, as well as at the *Mir29ab1* RE-ARE and non-specific regions. ENCODE data shows H3K4me2 marking the entire *Mir29ab1* locus, including the distal regions 2 kb away, and therefore can serve as a positive control for sequential IPs of the re-ChIP; *N* = 3. Bars indicate mean ± SEM; *P < 0.05, **P < 0.01 (paired *t*-test). (**H**) Structural model of Nrf2 and p63 binding to the DNA of the mouse *Mir29ab1* RE-ARE. Colors of the model of the core nucleosome binding as in (D).

Next, we analyzed the upstream and the downstream sequence of the *MIR29AB1* gene. Enrichment of AREs within ∼200 bp regions bound by p63 and covered by H3K27ac marks, a marker for active chromatin, has been reported in primary human keratinocytes (15). We found several new, and confirmed previously reported p53/p63 DNA-binding sites in promoter/enhancer regions of the mouse *Mir29ab1* and human *MIR29AB1* genes (13). Two RE-ARE pairs were identified in the proximity of the human *MIR29AB1* gene (Figure 3A). The upstream RE-ARE is part of an NRF2-bound enhancer 1 (Figure 3A; 13), and p63 strongly bound to this RE-ARE (Figure 3B). The distal downstream enhancer 2 (Figure 3A) had been detected by two independent p63 ChIP-seq analyses (15,25). In a tandem immunoprecipitation of the chromatin with p63 and NRF2 antibodies (re-ChIP), we found simultaneous binding of p63 and NRF2 to the proximal upstream RE-ARE (Figure 3C). The binding to the proximal upstream RE-ARE was further enhanced by SFN.

The close proximity of REs and AREs on miR-29 coding genes allowed us to model the interaction of p63 and NRF2 on the *MIR29AB1* upstream RE-ARE in the context of a nucleosome (Figure 3D). The structure of the *C. elegans* ortholog of NRF2, Skn-1, was used instead of mammalian NRF2, because Skn-1 has a better-resolved structure and because the mammalian and *C. elegans* genes are highly conserved. This model predicts an indirect, DNA-mediated interaction of p63 and NRF2. A 96-bp spacer separating RE and ARE may be necessary to accommodate the ‘bulky’ p63 tetramer and a long protruding b-Zip domain of the obligatory NRF2-binding partner, a small MAF protein (Figure 3D).

Analysis of the murine *Mir29ab1* gene identified a full p63 RE within 42-bp distance from the Nrf2-bound ARE (Figure 3E). Using ChIP of nuclear lysates from the epidermis of transgenic mice expressing a constitutively active Nrf2 mutant (caNrf2) (9), we detected binding of p63 to the *Mir29ab1* RE-ARE (Figure 3F). p63 binding was also detected in mouse epidermis expressing a dominant-negative Nrf2 mutant (dnNrf2), which abrogates the transcriptional activity of wild-type Nrf2 by occupying the AREs (32). The binding of p63 to the *Mir29ab1* RE-ARE is therefore independent of the transcriptional activity of Nrf2. Re-ChIP demonstrated a significant enrichment of the p63-Nrf2-bound *Mir29ab1* RE-ARE compared to a region void of both RE and ARE (Figure 3G). These results demonstrate simultaneous recruitment of p63 and Nrf2 to the *Mir29ab1* gene. Similar to the human RE-ARE, modeling of a 42 bp spacer DNA between the mouse *Mir29ab1* RE and ARE on a nucleosome demonstrated a possibility of simultaneous binding of Nrf2 and p63 (Figure 3H). The high conservation of the p63 and NRF2 interaction on the mouse and human genes points to a strong functional relevance.

### The DNA-binding and SAM domains of p63 and the transactivation domain of NRF2 are required for co-activation of the RE-ARE

**Figure 4.**
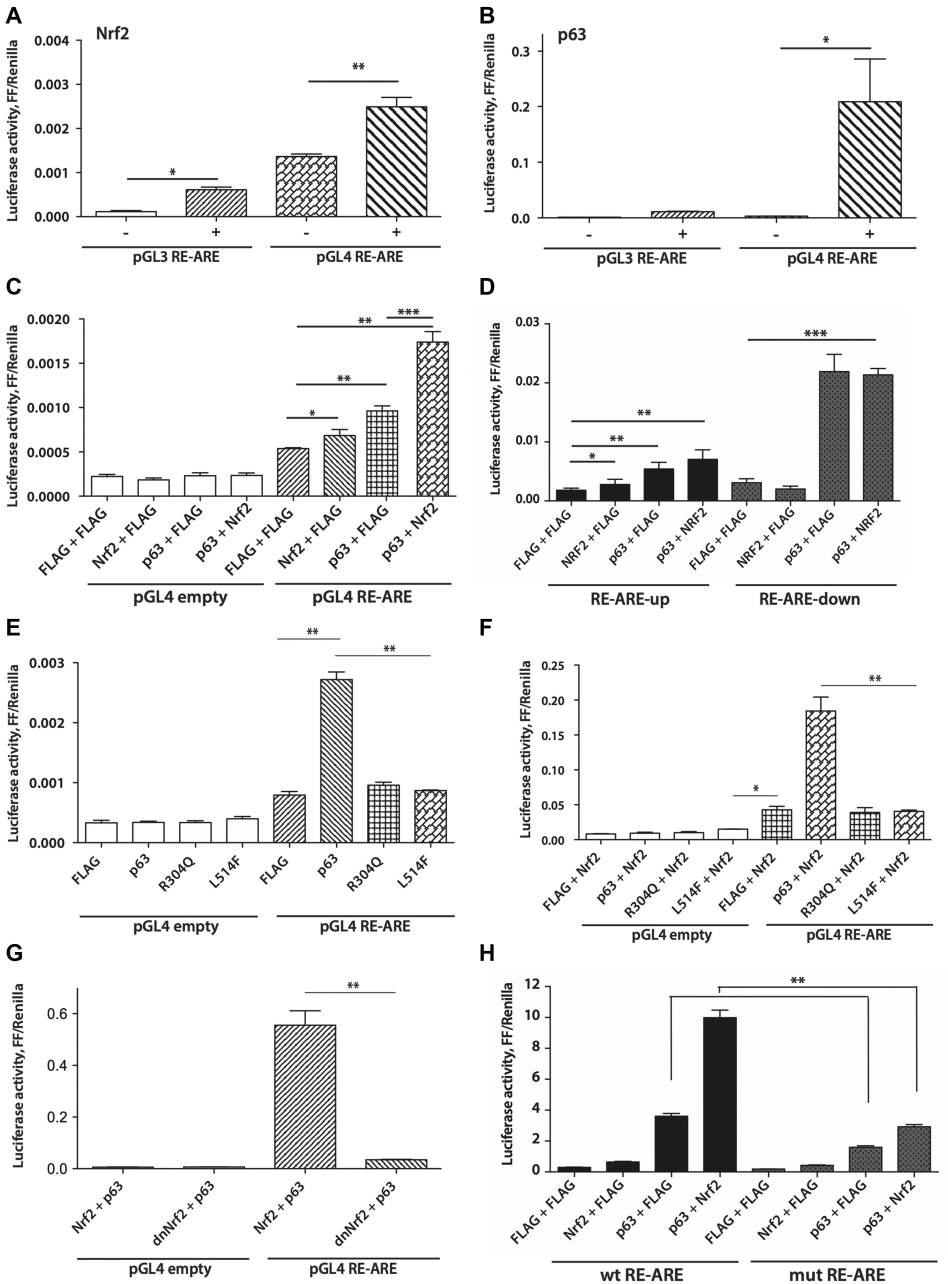
Transcriptional activity of NRF2 and p63 is required to co-regulate RE-AREs. (**A** and **B**) The RE-ARE from the murine *miR29ab1* gene was cloned into the pGL3 and pGL4 reporter vectors, and Firefly (FF) versus Renilla luciferase activity was measured 48 h following transfection of HEK 293T cells with pGL3 RE-ARE or pGL4 RE-ARE plasmids with or without an NRF2 expression plasmid. (**C**) Cells were co-transfected with NRF2 and/or p63 expression vectors or empty vector, combined with empty pGL4 or pGL4 RE-ARE. Cells were analyzed 48 h after transfection for luciferase activity. Empty vectors served as controls. (**D**) Human RE-ARE sequences indicated in Figure 3A were cloned into the pGL4 reporter vector and used for the co-transfection of HEK 293T cells. p63 and/or NRF2 expression vectors were added in the same transfection. Luciferase activity was measured 48 h following transfection. (**E** and **F**) Expression vectors for wt p63 and mutant R304Q and L514F p63 were used for the co-transfection of HEK 293T cells with empty pGL4 or pGL4 *Mus Mir29ab1* RE-ARE without or with an NRF2 expression vector. Luciferase activities were measured 48 h after transfection. (**G**) p63 and NRF2 (wt) or dnNRF2 expression vectors were used for the co-transfection of HEK 293T cells with empty pGL4 or pGL4 containing *Mus Mir29ab1* RE-ARE, and luciferase activities were measured. (**H**) Site-directed mutagenesis was performed to generate a mutated sequence lacking two core nucleotides essential for NRF2 binding (substituting GTCA to GTgt, see Figure 3E) in pGL4 RE-ARE. Cells were transfected with either mutant RE-ARE (mut RE-ARE) or *Mus* wt RE-ARE pGL4 reporter plasmids, and luciferase activity was measured under the same conditions upon transfection with p63 and/or NRF2 expression vectors; *N* = 3 biological replicates, *n* = 3 technical replicates. Bars indicate mean ± SEM; *P < 0.05, **P < 0.01, ***P < 0.001 (unpaired *t*-test).

Since both NRF2 and p63 function as transcriptional activators (33,34), we investigated if the activation of RE-AREs requires their joint transcriptional activity. This was tested in luciferase reporter assays using a fragment of the murine *Mir29ab1* gene containing the RE-ARE cloned into an empty reporter vector (pGL3) or into a reporter vector with a minimal promoter, but lacking an enhancer element (pGL4). Overexpression of NRF2 alone activated RE-AREs in both reporters (Figure 4A). The presence of the minimal promoter resulted in a stronger enhancer-like function of the RE-ARE and was required for activation by p63 (Figure 4B). Simultaneous overexpression of NRF2 and p63 further enhanced activation of the *Mir29ab1* RE-ARE, and we detected a significant synergistic effect (Figure 4C). Similarly, overexpression of p63 and NRF2 resulted in a synergistic activation of the human *MIR29AB1* proximal RE-ARE, whereas the downstream RE-ARE was predominantly activated by p63 (Figure 4D). Overexpression of NRF2 and p63 also lead to an increase in the endogenous primary miR-29ab1 transcript (Supplementary Figure S3D). Next, we tested whether DNA binding and transcriptional activity of p63 are required for the activation of the RE-ARE-containing enhancer. The DNA-binding mutant R304Q and sterile alpha motif (SAM)-domain mutant L514F of p63 (35,36) failed to activate the RE-ARE (Figure 4E) and abrogated the synergistic effect with NRF2 (Figure 4F). In addition, dnNRF2 abrogated the synergistic NRF2–p63-mediated activation of the RE-ARE (Figure 4G). Therefore, the transcriptional activity of p63 and NRF2 and the DNA-binding activity and SAM-domain of p63 are necessary to co-activate RE-AREs. A mutation in the NRF2-binding site of the RE-ARE (Figure 3E) decreased both p63-mediated activation and the synergistic effect of p63-NRF2 on the RE-ARE (Figure 4H), further supporting a co-dependence of NRF2 and p63 in activation of regulatory DNA elements.

### Synergism in NRF2–p63 transcriptional activity promotes expression of genes involved in keratinocyte proliferation

To identify targets of NRF2 and p63 in the epidermis genome-wide, we performed NRF2 ChIP-seq using lysates of primary human keratinocytes. While many of the called 3875 NRF2 peaks did not contain a classical ARE and remain to be characterized in the future, the well-characterized ARE of the NADP(H) dehydrogenase quinone 1 (*NQO1*) gene and also the *MIR29AB1* RE-AREs showed NRF2 binding, consistent with our previous results (Supplementary Figure S4) (13). Treatment of the cells with low concentrations of SFN induced a remarkable shift in binding of NRF2 to the DNA within 3 h (Supplementary Figure S5A).

When overlapped with H3K27ac- and p63-binding sites detected in primary keratinocytes (16), NRF2 bound mostly to enhancer regions, as expected. However, following activation with SFN, the NRF2-binding sites that overlapped with H3K27ac and p63 peaks were surprisingly clustered in promoter regions (Supplementary Figure S5B).

**Figure 5.**
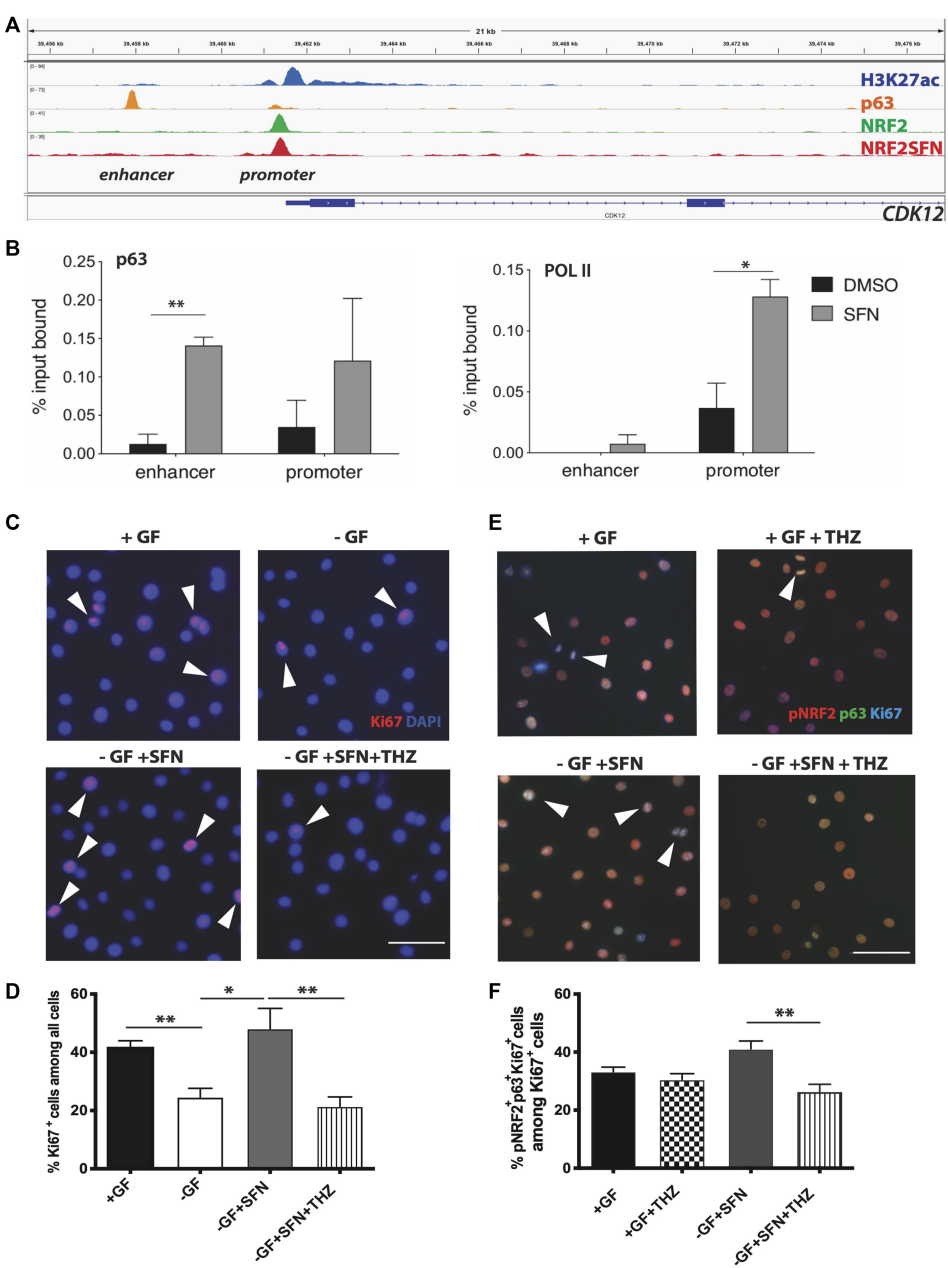
Stabilization of NRF2 activates the *CDK12* promoter and promotes keratinocyte proliferation. (**A**) Binding of H3K27ac, p63, baseline NRF2 and SFN-activated NRF2 to the *CDK12* gene in human keratinocytes as detected by ChIP-seq (15,16). (**B**) Enhanced p63 and Pol II binding to the *CDK12* gene in the regions indicated in (A) following SFN treatment (3 h, 5 μM); *N* = 3. Bars indicate mean ± SEM. *P < 0.05, **P < 0.01 (paired *t*-test). (**C–F**) Human keratinocytes were treated with SFN or vehicle in the presence or absence of the CDK12 inhibitor THZ531 and stained for Ki67 (red), counterstained with DAPI (blue) in (C) or for pNRF2 (red), p63 (green) and Ki67 (cyan) in (E). Note the increase in triple positive cells in the presence of SFN and their reduction by THZ531. (D) Quantification of Ki67^+^ cells among all cells in the field or (F) pNRF2^+^p63^+^Ki67^+^ cells among Ki67^+^ cells demonstrate the specific effect of THZ531 on proliferation of NRF2^+^p63^+^ cells. At least three fields with 50–100 cells were quantified in biological triplicate (*N* = 3). Bars indicate mean ± SEM. *P < 0.05, **P < 0.01 (unpaired *t-*test); scale bar: 50 μm.

By applying stringent criteria (highest number of the reads mapped to an annotated gene), we selected three candidate genes for a follow-up analysis. These genes encode cyclin-dependent kinase 12 (CDK12), a cell cycle regulator (37), BTB domain containing 10 (BTBD10), an activator of AKT kinase (38), and Family With Sequence Similarity 200 Member B (FAM200B), a protein with predicted DNA-binding function (GeneCards and UniProtKB: P0CF97). Unlike the ‘classical’ NRF2 targets *NQO1* and *PRDX1* (encoding peroxiredoxin 1), the regulatory regions of the three p63-NRF2 target genes contain a p63-bound enhancer within a relatively short distance from the TSS (0.5–3.5 kb; Figure 5A and Supplementary Figure S4B). The p63 peak in the NRF2-bound promoter region suggests an interaction between the p63-bound enhancer and the NRF2-bound promoter via chromatin looping.

The NRF2–p63 interaction is of likely functional relevance, since expression of all three genes significantly increased upon treatment with SFN (Supplementary Figure S6A). Interestingly, the mRNA of CDK12 increased significantly only briefly at 3 h of SFN treatment compared to a more stable increase in *NQO1* and *PRDX1* expression (Supplementary Figure S6B). It is possible that SFN activates a transient interaction between the p63-bound enhancer and a NRF2-bound promoter, resulting in augmented transcription of *CDK12*. To test this possibility, we used ChIP-qPCR with antibodies against p63 and polymerase II (POL II) on one of the enhancer–promoter pairs upstream of the *CDK12* gene (Figure 5B). SFN treatment significantly increased the binding of p63 to the enhancer and POL II binding to the promoter of the *CDK12* gene (Figure 5B), supporting a function of p63 in co-activating an NRF2-bound promoter.

The combined basal activity of p63 and NRF2 also control *CDK12* expression, since simultaneous siRNA-mediated knockdown of NRF2 and p63 caused a significant reduction in *CDK12* expression, while this was not the case in the single knockdown of either NRF2 or p63 (Supplementary Figure S6C).

### Activation of NRF2–p63 interactions promotes keratinocyte proliferation

CDK12 has crucial functions in gene expression, proliferation and genome stability, and is a target for anti-cancer therapy (37). We hypothesized that the activation of the *CDK12* enhancer is part of the joint NRF2–p63 function in proliferation-competent keratinocytes suggested by our *in silico* analysis (Figure 1). To test this possibility, we treated cells with the CDK12 inhibitor THZ531 (39), and found a significant reduction of keratinocyte proliferation as determined by analysis of the number of cells positive for the Ki67 proliferation marker (Supplementary Figure S6D and S6E). This difference was mild in growth factor-treated cells, but became statistically significant in the absence of growth factors.

To test if the NRF2–p63 interaction promotes keratinocyte proliferation via CDK12, we treated the cells with SFN and THZ531. In the absence of exogenous growth factors, addition of SFN promoted keratinocyte proliferation, and this was abrogated in the presence of THZ531 (Figure 5C and D). Importantly, THZ531 reduced not only the total number of Ki67-positive cells, but also a subpopulation simultaneously positive for pNRF2, p63 and Ki67 (Figure 5E and F). This result strongly suggests that NRF2–p63-activated transcription regulates proliferation at least in part via CDK12.

**Figure 6.**
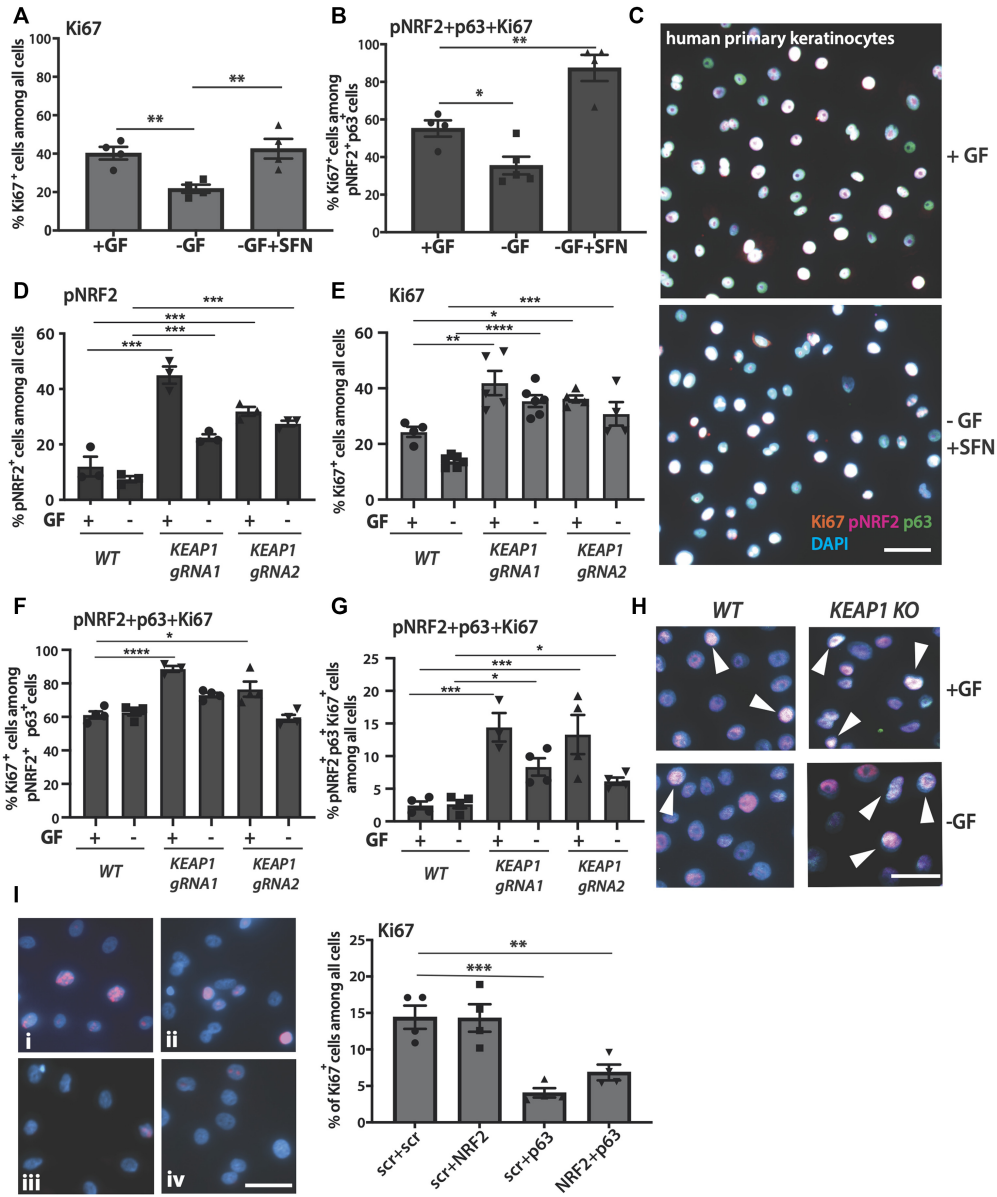
Enrichment of proliferating cells among pNRF2-p63 double-positive keratinocytes. (**A–C**) Human primary keratinocytes were cultured in the absence of growth factors (-GF) and treated for 3 h with vehicle (DMSO) or SFN and immunostained with antibodies against Ki67, pNRF2 and p63. Cells cultured in the presence of growth factors (+GF) were used as a control. (**A**) Percentage of Ki67^+^ cells among all cells. (**B**) Percentage of triple-positive cells among all pNRF2^+^p63^+^ cells. (**C**) Representative images from +GF and -GF+SFN cells with high numbers of Ki67^+^pNRF2^+^p63^+^ triple-positive cells (appear almost white); scale bar: 50 μm; *N* = 4. (**D–H**) Human primary keratinocytes were transduced with lentiviruses allowing CRISPR/Cas9-mediated *KEAP1* knockout using two different guide RNAs or control virus (*n* = 3). They were cultured for 3 h in the presence (+GF) or absence of growth factors (-GF) and stained for p63, pNRF2 and Ki67. (D) Percentage of pNRF2^+^ cells among all cells. (E) Percentage of Ki67^+^ cells among all cells. (F) Percentage of triple-positive cells among all pNRF2^+^p63^+^ cells. (G) Percentage of Ki67^+^pNRF2^+^p63^+^ among all cells. (H) Representative images from the wt control and KEAP1 gRNA lentivirus-transduced cells with Ki67^+^pNRF2^+^p63^+^ triple-positive cells (appear almost white, pointed with arrowheads). Cells were cultured in the presence (+GF) or absence (-GF) of growth factors. Ki67^+^ cells are labeled red, pNRF2^+^ cells far-red (appear magenta) and p63^+^ were green. Nuclei were counterstained with DAPI (blue); scale bar: 50 μm. (**I**) Primary human keratinocytes were cultured in 2D under exponential growth conditions, transfected with scrambled (i), NRF2 (ii), p63 (iii) or NRF2+p63 siRNAs (iv) and stained for Ki67. The percentage of Ki67^+^ cells was quantified. Bars indicate mean ± SEM. *P < 0.05, **P < 0.01, ***P < 0.001 and ****P < 0.0001 (one-way ANOVA).

In agreement with the enhanced NRF2–p63 interaction detected in nuclei of keratinocytes by PLA (Figure 2C–G), Ki67^+^ and pNRF2^+^p63^+^Ki67^+^ cells were significantly increased by SFN in cells cultured in the absence of growth factors (Figure 6A and B). As expected, withdrawal of growth factors significantly suppressed proliferation, but SFN treatment restored the number of proliferating keratinocytes to the levels seen in the presence of growth factors (Figures 5D and 6A). As a consequence, almost all pNRF2^+^p63^+^ cells were also Ki67^+^ (Figure 6B and C; Supplementary Figure S7). These results suggest that pharmacological activation of NRF2 promotes its synergistic activity with p63, resulting in restoration of the proliferative capacity of keratinocytes.

Since SFN has NRF2-independent activities, we used CRISPR/Cas9-mediated knockout (KO) of the NRF2 inhibitor KEAP1 to stabilize/activate NRF2 in primary human keratinocytes. KEAP1 KO indeed increased the percentage of pNRF2-positive cells in the nucleus and overall NRF2 levels (Figure 6D and Supplementary Figure S6F). Importantly, this was associated with a significant increase in cell proliferation, even in the absence of growth factors (Figure 6E), demonstrating that genetic activation of NRF2 mimics the effect of SFN.

The majority of KEAP1 KO pNRF2^+^p63^+^ cells were also positive for Ki67, and the number of pNRF2^+^p63^+^Ki67^+^ keratinocytes was significantly higher in KO compared to WT cells (Figure 6F–H). The increase in proliferation of KEAP1 KO pNRF2^+^p63^+^ cells was significant only in the presence of growth factors (Figure 6F); however, pNRF2^+^p63^+^Ki67^+^ keratinocytes significantly increased in total numbers in KEAP1 KO cells in the presence or absence of growth factors (Figure 6G and H).

While these results demonstrate that NRF2 activation promotes keratinocyte proliferation, knockdown of NRF2 alone and thus reduction of its basal activity had no effect on proliferation in 2D and under exponential growth conditions (Figure 6I). This is consistent with the normal keratinocyte proliferation in *Nrf2* knockout mice (6,40) and may result from compensation by other Nrf transcription factors. By contrast, siRNA-mediated reduction of p63 levels resulted in a strong decrease in keratinocyte proliferation, and additional knockdown of Nrf2 did not further enhance this effect (Figure 6I).

**Figure 7.**
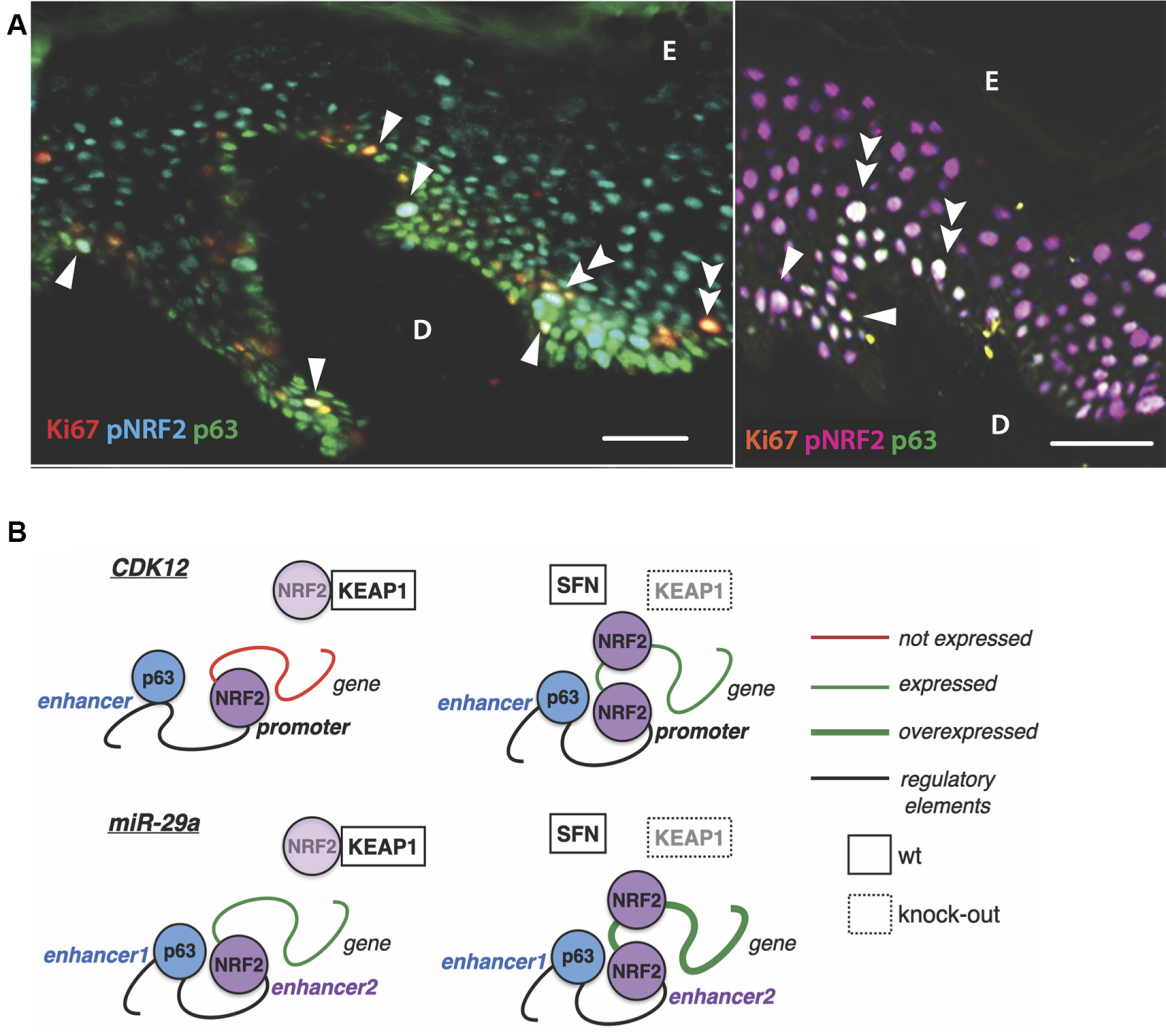
Co-localization of NRF2 and p63 in the human epidermis. (**A**) Frozen sections from human skin were stained with Ki67 (red), pNRF2 (cyan) and p63 (green) (left panel); and Ki67 (orange), pNRF2 (red) or p63 (green) (right panel). Ki67^+^pNRF2^+^p63^+^ triple-positive cells appear light yellow on the left panel and almost white on the right panel; scale bar: 50 μm. D: Dermis; E: Epidermis. Triple-positive keratinocytes of the basal layer are indicated with single arrowheads and those in the lower suprabasal layer with double-arrowheads. (**B**) Scheme showing effects of SFN and genetic activation of NRF2 on p63-mediated expression of *CDK12* and *miR29ab1*.

Finally, we determined the potential relevance of the NRF2–p63 cross-talk for the regeneration of the human epidermis. Interestingly, pNRF2^+^p63^+^ cells that also expressed Ki67 were found in the basal epidermal layer of foreskin (Figure 7A, left) or abdominal (Figure 7A, right) human skin (single arrowheads), and even in the lower suprabasal layers (Figure 7A, double arrowheads). This finding suggests that the interaction of both transcription factors may retain the proliferative capacity of keratinocytes that have already entered the differentiation program.

Taken together, our results unravel an unexpected cross-talk of NRF2 and p63 at the level of transcription, which defines populations of proliferating cells *in vivo* and is mediated by the preferential binding of NRF2 and p63 to the enhancers and promoters of their common target genes (Figure 7B).

## DISCUSSION

We discovered a physical interaction of two key regulators of epithelial function: p63 and NRF2. The *in vivo* relevance of this finding is suggested by the co-localization of pNRF2 and p63 in the nuclei of keratinocytes within the lower layers of human epidermis. Importantly, both proteins were co-expressed with Ki67 in a subset of basal and also suprabasal keratinocytes. Together with our p63/NRF2 gain-of-function studies performed *in vitro*, this finding suggests that the combined activity of both transcription factors is important to maintain the proliferative capacity of keratinocytes, including those that have already entered the differentiation program. A positive effect of NRF2/p63 on proliferation is further supported by the reduced expression of *CDK12* upon combined knockdown of both genes. However, knockdown of NRF2 in exponentially growing, non-differentiated cells in 2D cultures did not further suppress proliferation of keratinocytes with p63 knockdown and also did not have an effect on proliferation of keratinocytes with normal p63 levels. This is in contrast to previous studies, which showed that CRISPR/Cas9-mediated deletion of NRF2 reduced proliferation of human keratinocytes with high stem cell potential (41). Therefore, NRF2 activity may be particularly relevant for proliferation of stem cells. It is also possible that only a complete loss of NRF2 affects proliferation of human keratinocytes, and few molecules, which remain after siRNA-mediated knockdown, may be sufficient to maintain keratinocyte proliferation. Finally, NRF2 and its cross-talk with p63 could be particularly relevant for proliferation of suprabasal cells, which are already committed to differentiation as suggested by its predominant expression in these layers.

In addition to its effect on proliferation, the p63/NRF2 cross-talk may also promote the early differentiation process as suggested by the co-expression of both transcription factors and their combined target *MIR29AB1* in suprabasal, differentiated keratinocytes. Future studies should therefore address the effect of p63/NRF2 targets on the differentiation process.

In addition to its function in the normal epidermis, activation of p63/NRF2 may also have a positive effect on wound healing. Thus, genetic activation of Nrf2 in keratinocytes strongly promoted the proliferation of different stem cell populations during mouse wound healing (42), and pharmacological activation of the Nrf2 pathway accelerated wound healing in healthy and diabetic mice. Therefore, this approach may be beneficial for the treatment of chronic wounds (43,44). Our results suggest that combined activation of Nrf2 and p63 could be even more efficient, for example, through maintenance of the proliferative capacity of cells in the suprabasal layers of the wound epidermis.

Analysis of ChIP-Seq data performed in this study and published in the literature identified individual and common targets of both transcription factors, which changed upon chemical activation of NRF2 (45). Surprisingly, the number of NRF2-bound sites was reduced upon SFN treatment, suggesting that stabilization of NRF2 results in concentration of NRF2 binding to specific sites. Importantly, the simple presence of an ARE does not define these sites, indicating the requirement of other factors for the recruitment of NRF2 to specific loci, which remain to be identified.

An enrichment of AREs within ∼200 bp regions bound by p63 and covered by H3K27ac marks (15) suggests a role for p63 in the activation of NRF2-bound promoters. Consistent with this hypothesis, ChIP-seq analysis found AREs enriched within 100 bp distances from the center of p53-bound REs (46). Due to the longer spacer lengths between the RE and ARE motifs, we suggest that the type of interaction between NRF2 and p63 on RE-AREs is DNA-and nucleosome-mediated (47). We do not know if phosphorylation of NRF2 is required for its interaction with p63. Therefore, it will be interesting to address this question using an NRF2 variant with a point mutation in the S40 phosphorylation site.

NRF2 and p63 possibly interact via short-distance chromatin looping to regulate expression of *CDK12*. Interestingly, CDK12 activity was shown to regulate the transcription of certain NRF2 target genes, thereby controlling a switch in gene expression from a metabolically active state to a ‘stress-defense mode’ (48). Together with our data, this finding suggests that CDK12 provides a positive feedback for the activation of the NRF2 response. Our data also demonstrate that CDK12 activity in keratinocytes contributes to the enhanced proliferation observed upon activation of NRF2 and p63. The other common p63 and NRF2 targets that we identified in this study may further contribute not only to the promotion of keratinocyte proliferation, but also to aspects of differentiation, which should be tested in future studies. Finally, it will be important to identify compound combinations that activate both TFs for potential therapeutic applications and to identify the relevant downstream targets in different stratified epithelia.

## DATA AVAILABILITY

Input and IP ChIPseq files are deposited on GEO under accession number GSE150760. All other data are presented in the manuscript or in the Supplementary Files.

## Supplementary Material

gkab167_Supplemental_Files
