# Supplementary material for: Interaction of the NRF2 and p63 transcription factors promotes keratinocyte proliferation in the epidermis
Source: Nucleic Acids Res. 2021 Mar 25;49(7):3748–63. doi: 10.1093/nar/gkab167 (PMC8053124; doi:10.1093/nar/gkab167)
Supplement: gkab167_Supplemental_Files [file gkab167_supplemental_files.zip › Suppl.Fig.-corr.2.pdf]

Kurinna_Supplementary Figure 1

A

**NRF2 ARE:**
(A/G)**TGA(C/G/T)**(A/C/T)(A/C/T/G)A/G/T)**GC**(A/T)

**p63 RE:**
(A/G/T)**C(A/C/T)(A/T)G**(C/T)(C/T)(C/T)(A/C/T/G)(A/G/T)(A/G/T)**C(A/C/T)TG**(C/T)

NRF2 ARE: RTGABHNDGCW

p63 RE: CHWGYYYNDDCHTGY

**1. ARE inside p63 RE**

(opposite DNA strands)

*1671 sites*

CHWGCYYNDDCHTGY
--WCGRRWYAGTA--

**2. RE-AREs with shared nucleotides**

(0 to 10 nt)
*721 sites*

CHWGYYYNDDCHTGYRTGABHNDGCW

RTGABHNDGCWCHWGYYYNDDCHTGY

-GCHWGCTCATDCHTGY
WCGDWCGAGTA------

**3. RE-ARE with a spacer**

($n \geq 1$)
*88406 sites*

CHWGYYYNDDCHTGY-(N)n-RTGABWNDGCW

n < 7 bp : exclusive binding

7 > n < 121 bp : nucleosomal binding

n > 121 bp : long-range interaction

B

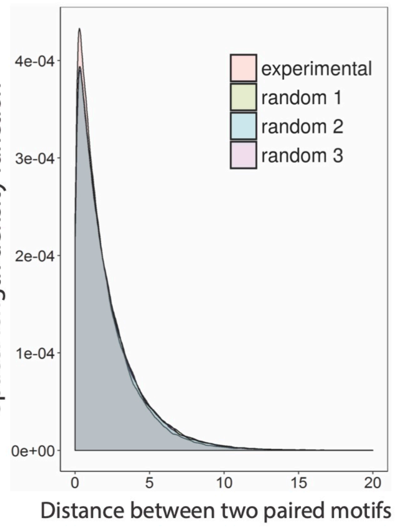


C

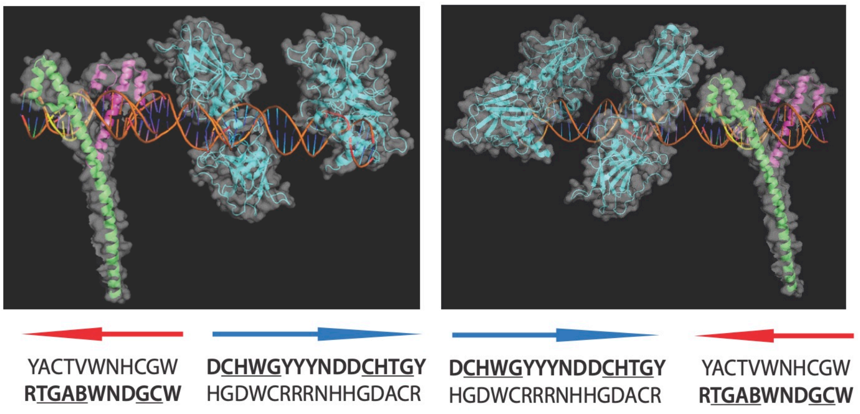

Kurinna_Supplementary Figure 2

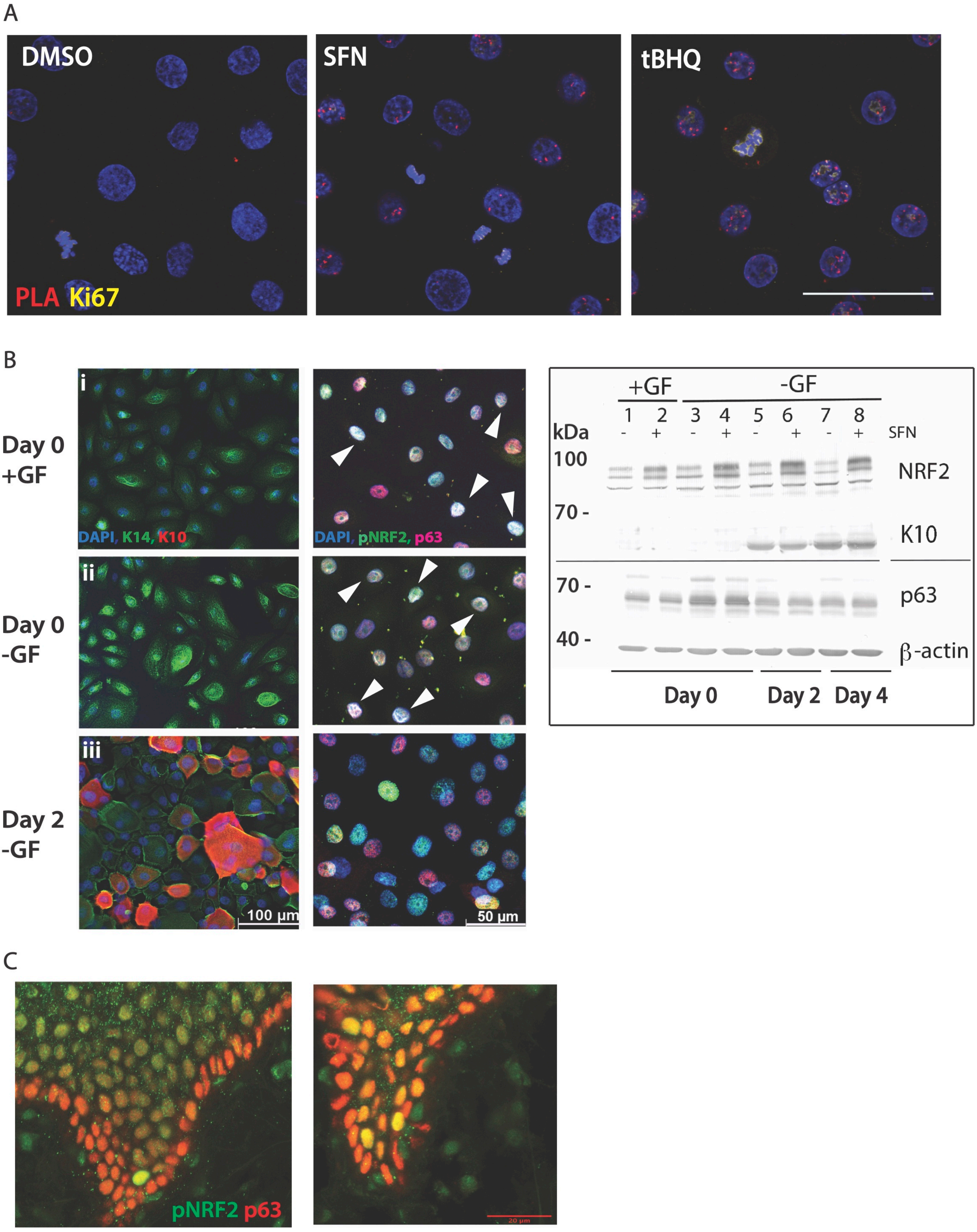

Kurinna_Supplementary Figure 3

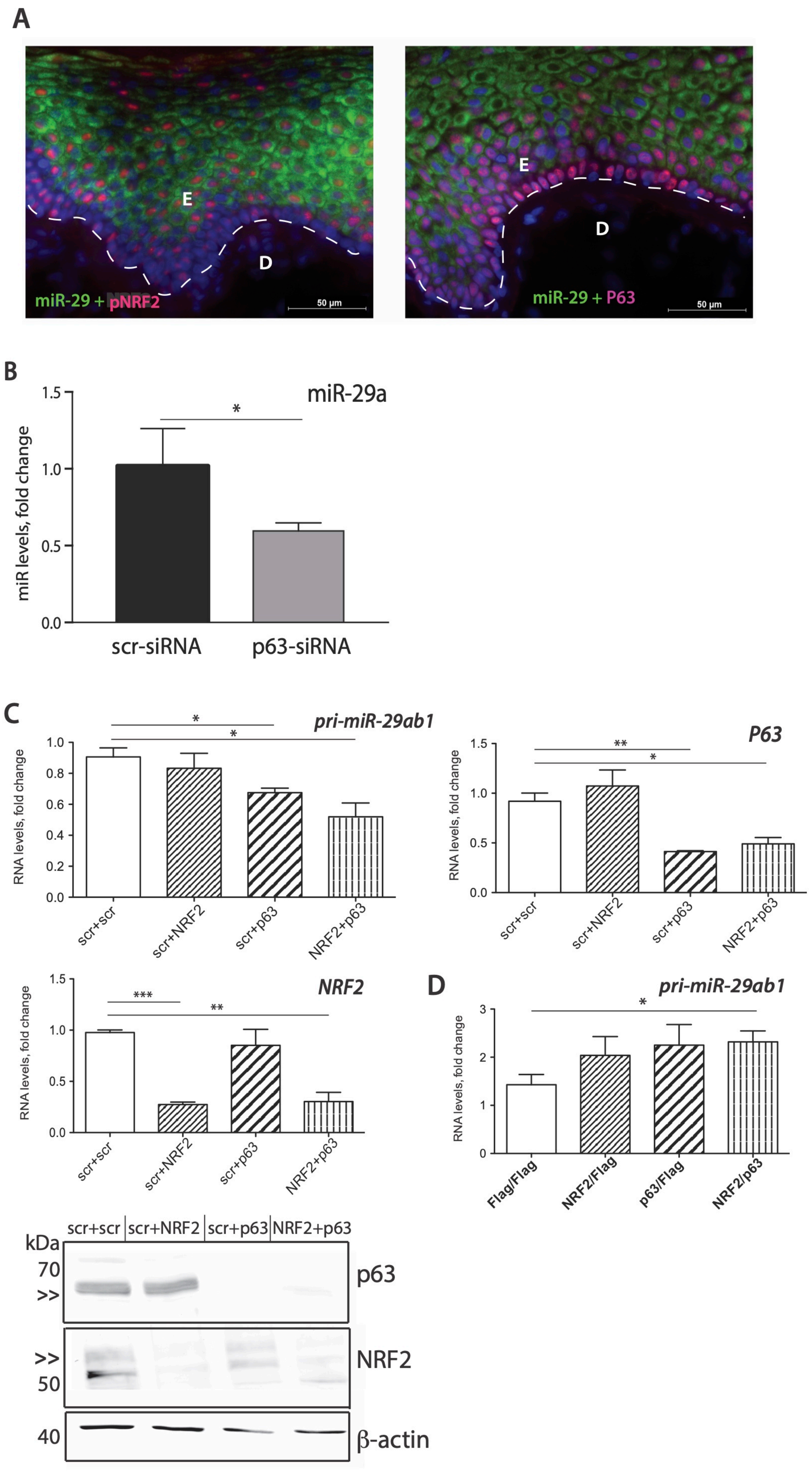

Kurinna_Supplementary Figure 4

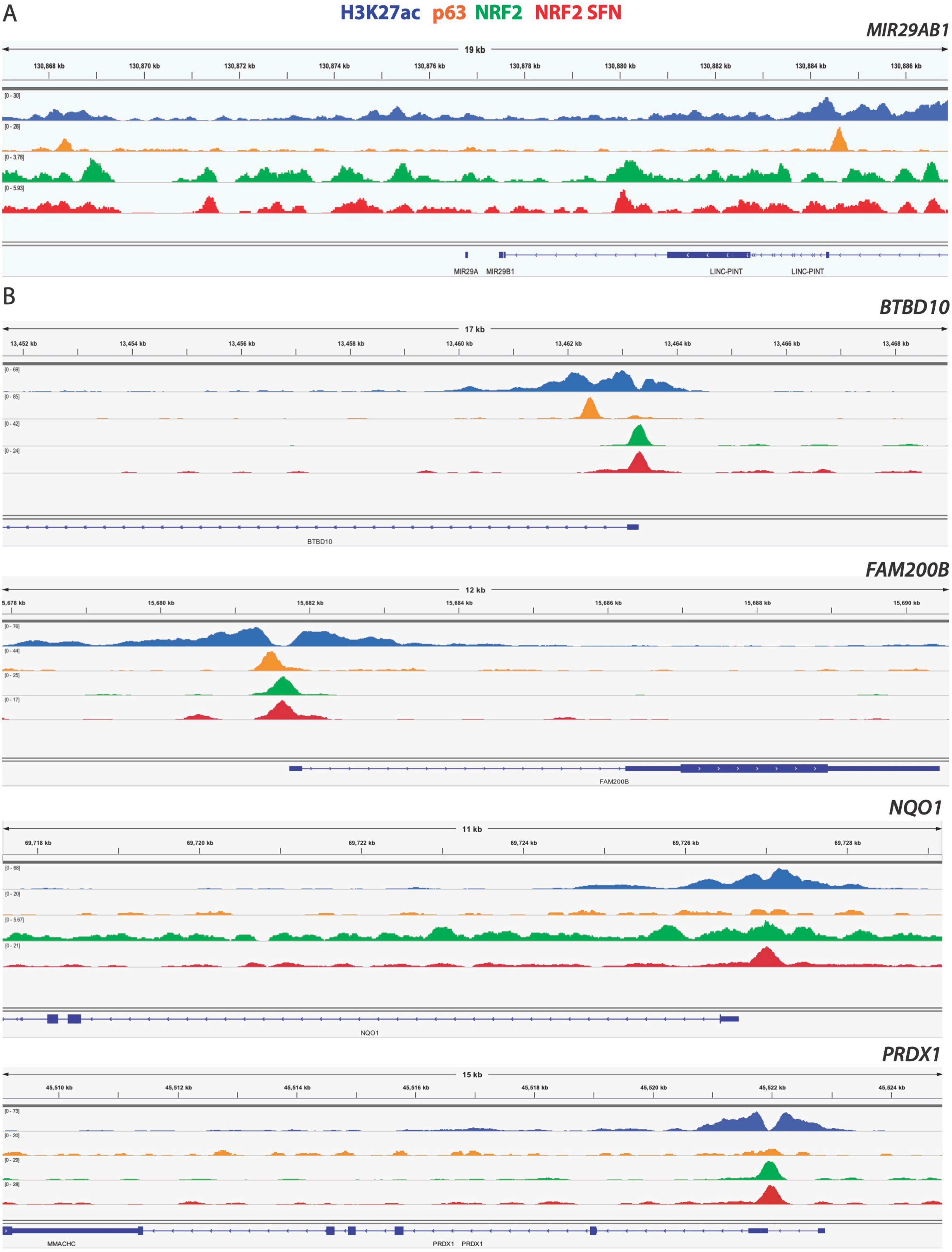

Kurinna_Supplementary Figure 5

A

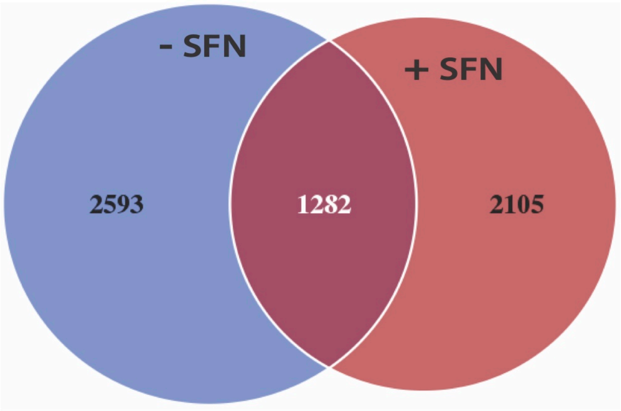


B

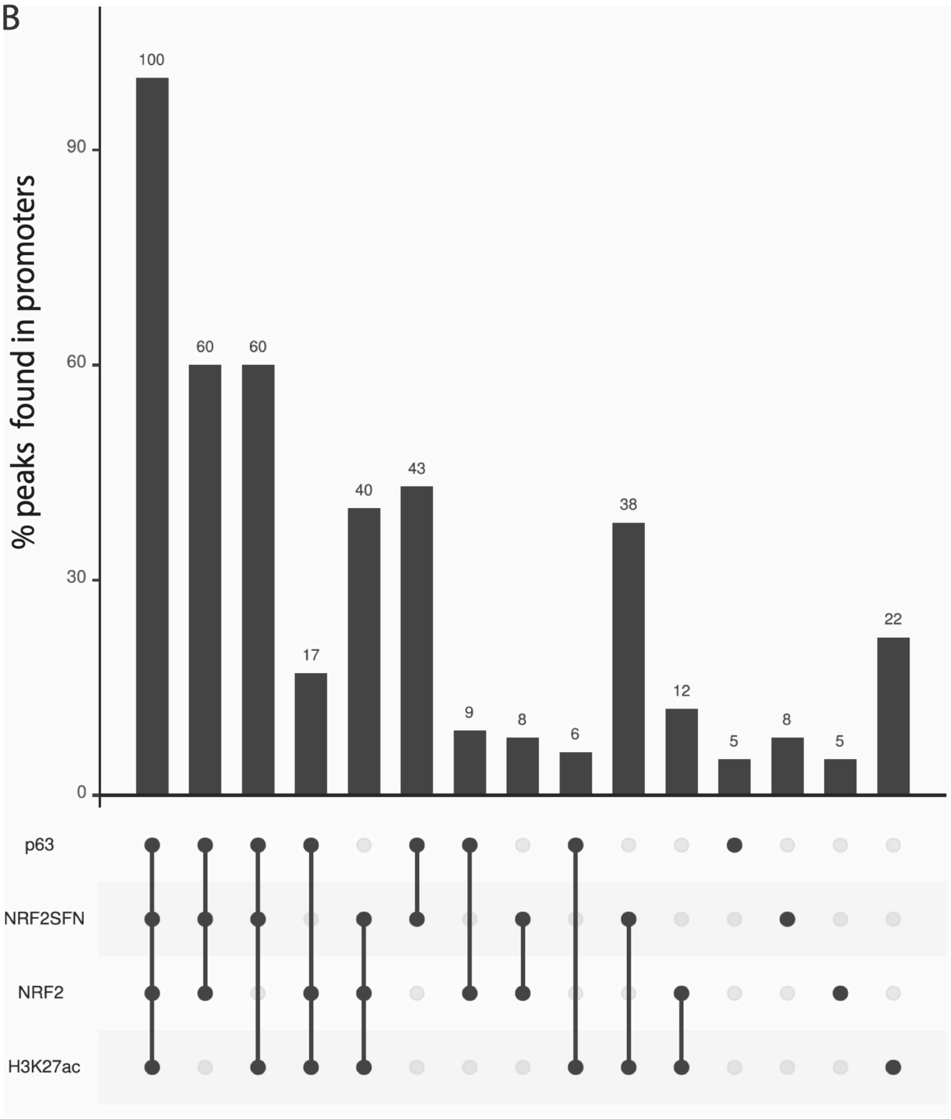

Kurinna_Supplementary Figure 6

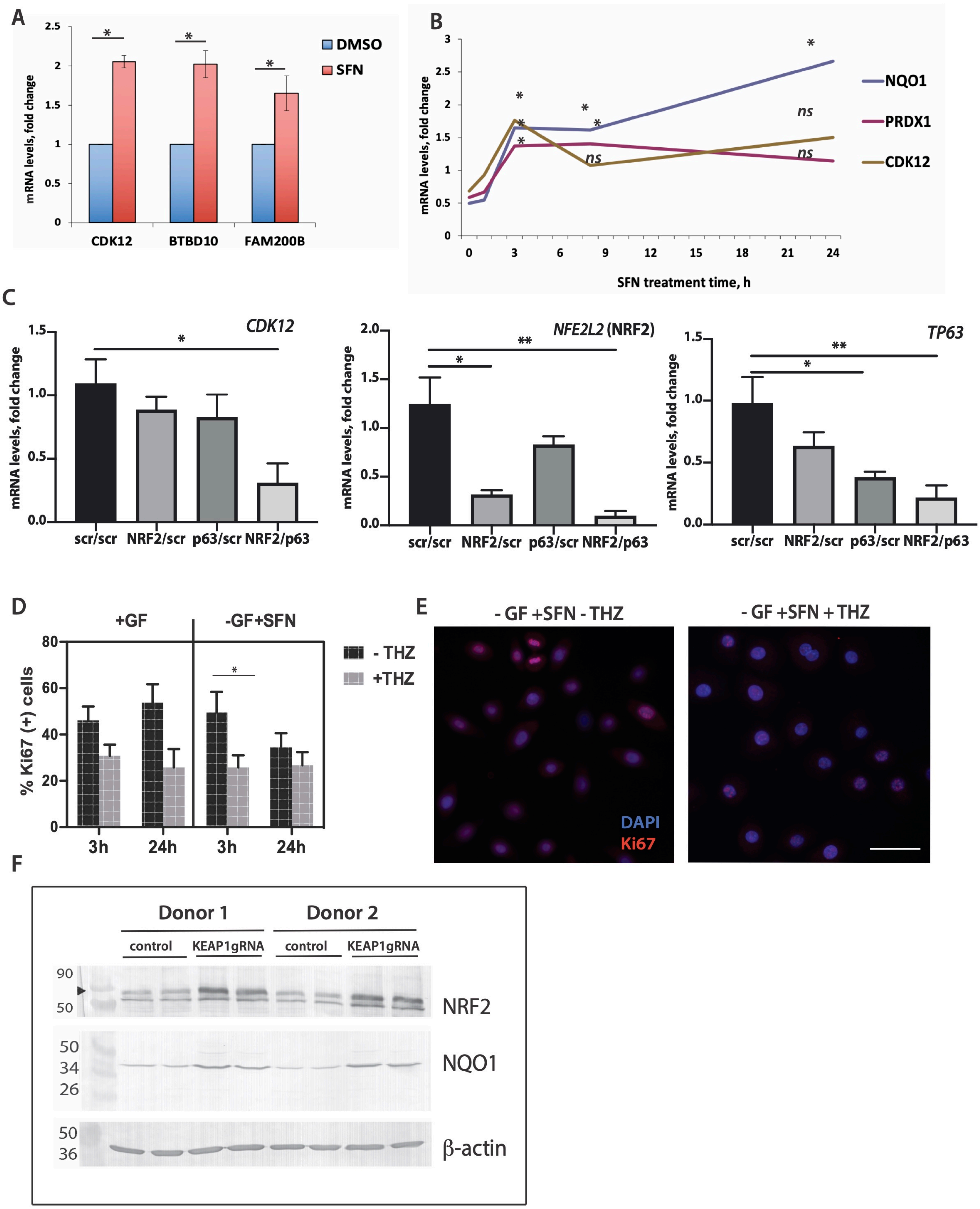

Kurinna_Supplementary Figure 7

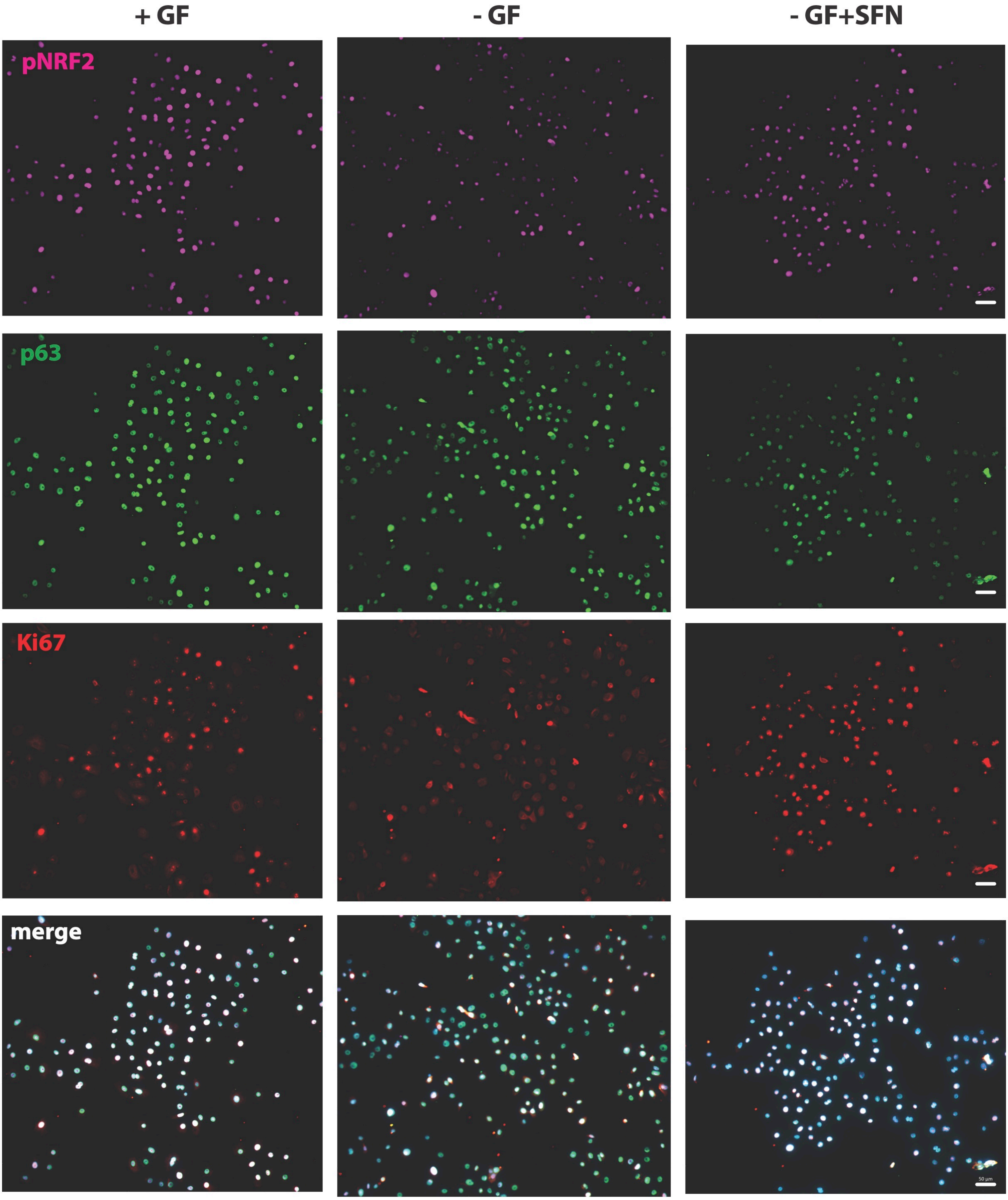

## Supplementary Figures

**Supplementary Figure S1. Genome-wide detection of RE-AREs.**

(A) The published NRF2 ARE and p63 RE sequences were coded using IUPAC nomenclature. The locations of p63 REs (CHWGYYYNDDCHTGY) and NRF2 AREs (RTGABHNDGCW) were extracted from data of a genome-wide analysis of human genome assembly GRCh37 using a Python script (Supplementary File S1). Examples of the combinatorial RE-ARE sequences are indicated, including (1) ARE inside RE, (2) RE-ARE with shared nucleotides and (3) RE-ARE with spacer. Core nucleotides of REs and AREs are in bold and underlined. The total number of RE-ARE pairs was calculated genome-wide using R script.

(B) Each p63 RE found in the genome was paired with the nearest ARE to compute the distances between p63 RE and ARE motifs in the experimental genome *vs*. distances between randomly distributed p63 RE and AREs (random). A total number of REs and AREs discovered genome-wide were distributed proportionally to the length of the chromosome as compared to the length of the genome and used as a control for non-random RE-ARE distance distribution (Supplementary File S2. Partial overlay of three randomized controls and the experimental density functions appear in grey, showing an enrichment of the shortest distance between paired RE and ARE motifs in the experiment (peak in light red).

(C) DNA-binding domains of small MAF proteins (green ribbons), Skn-1 (magenta ribbons), and p63 (blue ribbons and beta-sheets) were used for the modelling of NRF2-p63 interactions on RE-ARE sequences (DNA backbone, orange) in PyMOL v1.3. Two orientations for the pairs (head-to-tail and head-to-head) are shown as examples.

**Supplementary Figure S2. Co-localization of NRF2 and p63 in human epidermis and in cultured keratinocytes is promoted by pharmacological NRF2 activation.**

(A) Representative proximity ligation assays (PLA) using antibodies for pNRF2 and p63, counterstained with an antibody against Ki67 (appear light yellow in mitotic cells). PLA staining (red) is visible in resting and mitotic keratinocytes treated with SFN or tBHQ. Scale bar: 50 µm.

(B) Immunofluorescence staining of primary keratinocytes cultured in growth factor containing (+GF) medium at subconfluency (i), at confluency immediately following GF withdrawal (ii) and after 4 days of growth factor withdrawal (-GF, iii). Addition of GF in keratinocyte medium maintains them in an undifferentiated, basal-like state. Withdrawal of GFs activates early differentiation as revealed by expression of the differentiation marker cytokeratin 10 (K10). Left panel: expression of K14 (marker for non-differentiated cells) and K10; middle panel: nuclear staining for pNRF2 and p63. Scale bars: 100 µm (left panel) and 50 µm (middle panel). Right panel: Western blot using antibodies against p63 and NRF2 and total cell lysates from keratinocytes cultured as described above, and in addition treated with 5 µM SFN or vehicle (DMSO). K10 was used to monitor differentiation; β-actin was used as a loading control.

(C) Confocal images of immunofluorescence staining of human skin sections for pNRF2 (green) and p63 (red). Scale bar: 20 µm. Keratinocytes double-stained for pNRF2 and p63 appear in yellow and are located in basal and suprabasal epidermis of rete ridges.

**Supplementary Figure S3. NRF2 and p63 co-regulate expression of miR-29a in human keratinocytes.**

(A) Immunofluorescence staining of human skin sections for pNRF2 (magenta; left panel) or p63 (magenta; right panel) combined with miR-29 *in situ* hybridization (green). Bars: 50 µm.

(B) Human keratinocytes were transfected with scrambled or p63 siRNAs as indicated, and levels of the primary miR-29ab1 transcript were measured by qRT-PCR after 72 h of incubation. Expression levels in cells transfected with scr siRNA were set to 1.

(C) Human keratinocytes were transfected with scrambled, p63 and/or NRF2 siRNA and analysed

for pri-miR-29ab1, P63 and NRF2 RNA by qRT-PCR. N = 3, n = 3. The lower panel shows representative Western blot confirming efficient knock-down of p63 and NRF2.

(D) HEK 293T cells were transfected with empty vector (Flag), or with expression constructs for deltaNp63α and/or NRF2. Levels of pri-miR-29ab1 transcripts were measured by qRT-PCR after 24h of incubation. N = 3, n =3.

Bars indicate mean +/- SEM. *p < 0.05; **p < 0.01, ***p < 0.01 (unpaired t-test).

**Supplementary Figure S4. NRF2 and p63 activity on regulatory elements of target genes.**

Representative snapshots of the peak analysis from the IGV genome browser showing H3K27ac (blue), p63 (orange), NRF2 (green), and NRF2-SFN (red) ChIP-seq data for the selected genes: (A) enhancers of *MIR29AB1* bound by NRF2 and p63 (B) Promoters of *BTBD10* and *FAM200B* bound by NRF2 with a p63 peak nearby. Please, compare to *CDK12* on Figure 5A. Control *NQO1* and *PRDX1* promoters are not bound by p63, and the *NQO1* promoter shows a significant increase in NRF2 binding following SFN treatment.

**Supplementary Figure S5. Identification of p63- and NRF2-regulated genes in keratinocytes by ChIP-seq.**

(A) Venn diagram representing the number of binding sites identified by NRF2 ChIP-seq in primary human keratinocytes treated with SFN or vehicle (-SFN; DMSO). Note that only about half of the sites are common between NRF2 at the basal state (blue) and upon NRF2 activation (red).

(B) Number of peaks for H3K27ac, p63, NRF2, and NRF2 activated with SFN (NRF2SFN) seen after ChIP-seq, individually or simultaneously found in close proximity to known gene promoters displayed as percentage of total number of identified peaks.

**Supplementary Figure S6. NRF2 activation promotes keratinocyte proliferation via CDK12.**

(A) Human keratinocytes were treated with SFN or vehicle (DMSO) for 3 h. Samples were analysed by qRT-PCR for expression of *CDK12*, *BTBD10*, and *FAM200B*.

(B) mRNA of *CDK12* and control genes *NQO1* and *PRDX1* were analyzed in HaCaT cells over the time course of treatment with 5µM SFN.

(C) Human keratinocytes were transfected with scrambled, p63 and/or NRF2 siRNAs and analysed for the mRNA of *CDK12, NRF2,* and *TP63* by qRT-PCR in three independent experiments.

(D, E) Primary human keratinocytes were cultured in the presence (+GF) or absence (-GF) of growth factors and treated with SFN for 3 h and in the presence or absence of THZ531 for 3 h or 24 h. Cells were analysed by immunofluorescence staining for Ki67. The percentage of $Ki67^+$ cells among all cells is shown. At least three fields with 50-100 cells were quantified in biological triplicate per treatment. N = 3. Representative stainings for Ki67 (red), counterstained with DAPI (blue) for the significant result are shown in (b). Scale bar 50 µm.

(F) Primary keratinocytes isolated from foreskin epidermis of two healthy donors (Donor 1 and Donor 2) were transduced with a lentivirus allowing CRISPR/Cas9-mediated *KEAP1* knock-out using two different guide RNAs. Cell lysates were analysed for KEAP1, NRF2, the NRF2 target NQO1 and β-actin (loading control).

Bars indicate mean +/- SEM. ns: non-significant; N = 3, n = 3. Bars indicate mean +/- SEM. *p < 0.05; **p < 0.01, two-way ANOVA.

**Supplementary Figure 7. SFN treatment enriches for pNRF2- and p63-positive proliferating keratinocytes.**

Primary human keratinocytes were cultured in the presence of growth factors (+GF) or treated with SFN or vehicle (DMSO) in medium without growth factors for 3 h (-GF and -GF+SFN, respectively). pNRF2, p63, and Ki67 single-positive cells were detected with the specific primary antibody, followed by the secondary fluorophore-coupled antibodies emitting in far red (false-

coloured as magenta, pNRF2), green (p63), and orange (Ki67). Triple-positive Ki67-pNRF2-p63 cells (merged) appear white. Nuclei were counterstained with DAPI (blue). Scale bar: 50 µm.
